# Ginsenoside Rd protects against acute liver injury by regulating the autophagy NLRP3 inflammasome pathway

**DOI:** 10.1038/s41598-025-87991-9

**Published:** 2025-01-28

**Authors:** Xiaomei Zhong, Yibin Sun, Yanxiang Lin, Shan Deng, Huan Wang, Xian Zhou, Jinjian Lu, Yanfang Zheng, Ruoyin Luo, Mingqing Huang, Jianyuan Song

**Affiliations:** 1 The Affiliated People’s Hospital, Academy of Integrative Medicine, Fujian University of Traditional Chinese Medicine, Fuzhou, 350108 China; 2 Kaifeng Hospital of Traditional Chinese Medicine, Kaifeng, 475000 China; 3 The Affiliated People’s Hospital, College of Pharmacy, Fujian University of Traditional Chinese Medicine, Fuzhou, 350108 China; 4 NICM Health Research Institute, Western Sydney University, Westmead, NSW 2006 Australia; 5 State Key Laboratory of Quality Research in Chinese Medicine, Institute of Chinese Medical Sciences, University of Macau, Avenida da Universidade, Taipa, Macao China; 6 School of Pharmacy and Pharmaceutical Sciences, Ulster University, Belfast, UK; 7 Department of Radiation Oncology, Fujian Medical University Union Hospital, Fuzhou, 350001 Fujian China

**Keywords:** Ginsenoside Rd, Acute liver injury, Autophagy, Inflammation, AMPK/mTOR/ULK1 pathway, Thioacetamide, Hepatic stellate cell line, Biochemistry, Biological techniques, Biotechnology, Cell biology, Molecular biology, Biomarkers, Gastroenterology, Medical research

## Abstract

Ginsenoside Rd (Rd) is a bioactive compound predominantly found in *Panax ginseng* C.A. Meyer and *Panax notoginseng* (Burkill) F.H. Chen ex C.H. Chow, both species belonging to genus Panax in the Araliaceae family. However, its hepatic protective effect against acute liver injury and related mechanistic action remain unexplored. To investigate the protective effect of Rd against thioacetamide (TAA)-induced acute liver injury and assess its underlying regulatory mechanisms related to autophagy and inflammation. Forty-eight 8 weeks old C57BL/6 mice were treated with saline (control or model group), Rd (12.5 mg/kg, 25 mg/kg or 50 mg/kg), and diammonium glycyrrhizinate (DG, 30 mg/kg) for three days. Then the mice were stimulated with TAA to establish acute liver injury model, excluding the control group. HSC-T6 cells were treated with Rd at concentrations of 2.5, 5, or 10 µM, for 12 h with or without Lipopolysaccharide (LPS) stimulation at 100 ng/mL. Immunofluorescence staining, qPCR and Western blot were employed to analyze the expressions of genes and proteins associated with inflammation and autophagy. To validate the role of Rd in regulating autophagy and inflammation, the autophagy inducers, rapamycin and GSK621, were utilised in reverse validation experiments in cells. Rd exhibited significant hepatic protective effects in mice by reducing the serum levels of Aspartate aminotransferase (AST), Alanine aminotransferase (ALT), Glutathione S-transferase (GST) and Lactate dehydrogenase (LDH) with acute liver injury. It exhibited strong anti-inflammatory effect by reducing inflammation associated protein, such as cyclooxygenase-2 (COX-2), inducible nitric oxide synthase (iNOS), nod-like receptor protein 3 (NLRP3), associated speck-like protein containing a CARD (ASC), interleukin-18 (IL-18) and interleukin-1β(IL-1β) proteins and the mRNA expression levels of COX-2, Tumor Necrosis Factor α (TNF α), interleukin-6 (IL-6) and iNOS were decreased in liver tissue. And Rd inhibited LPS-induced inflammation by reducing the expression of COX-2 and NLRP3 in HSC-T6 cells. Moreover, not only in vivo but also in vitro, Rd downregulated the expression of LC3II, Beclin1, phosphorylation-AMP-activated protein kinase (p-AMPK), phosphorylation-ULK1 (p-ULK1) and upregulated the expression of p62 and phosphorylation-mechanistic target of rapamycin (p-mTOR) to suppress autophagy via the AMPK/mTOR/ULK1 pathway. Finally, the inhibitory effects of Rd on autophagy and inflammation in HSC-T6 cells were partially blocked by rapamycin and GSK621. Rd is a promising therapeutic agent to protect liver against TAA-induced acute liver injury by regulating the autophagy-NLRP3 inflammasome pathway.

**Supplementary Information:**

The online version contains supplementary material available at 10.1038/s41598-025-87991-9.

## Introduction

The liver is an organ with a unique immune system, comprised of hepatocytes, sinusoidal cells, and perisinusoidal cells. Acute liver injury (ALI), which includes drug-induced, chemical-induced, and immune-mediated liver injury in clinical classifications, can lead to liver dysfunction^1^. Long-term liver injury can lead to hepatic fibrosis, cirrhosis, and even life-threatening conditions such as liver failure and liver cancer. Therefore, preventing and treating liver injury is determinant in halting its progression, thus emphasizing the fundamental significance of developing hepatoprotective therapeutic agents.

Chemical-induced liver injury is a prevalent form of liver damage, and in studies aiming to induce liver injury models, substances such as TAA, carbon tetrachloride (CCl4), and D-galactosamine are often utilized^2^. TAA is a hepatotoxic compound that undergoes metabolism by CYP450 enzymes, leading to the formation of TAA-sulfur oxide within the body. This metabolic process disrupts hepatic metabolism, impacting proteins and lipids, and triggering oxidative stress^3^. Notably, the activation of autophagy in hepatic stellate cells (HSC) plays a critical role in TAA-induced liver injury and fibrosis^4^.

Abnormal autophagy levels can significantly influence the progression of liver diseases. Autophagy, a self-defense mechanism in the body, degrades damaged cellular organelles and recycles biomolecules to prevent cellular damage and dysfunction^5^. This process is influenced by various factors such as stress, inflammation, and apoptosis, serving as a means of cellular self-protection under oxidative stress conditions^6^. However, excessive autophagy can induce pathological changes in tissues^7^. Indeed, while moderate autophagy eliminates damaged organelles and proteins, excessive autophagy can exacerbate liver diseases^8^ and promote hepatic stellate cell activation, thereby worsening liver fibrosis^9^. This indicates that autophagy plays a dual role in liver diseases.

Regulating autophagy has been demonstrated to alleviate liver injury, highlighting its potential as a significant therapeutic approach for various liver diseases^10,11^. This discovery opens opportunities to explore the regulatory effects of drugs on autophagy to identify potential treatments for liver injury. Extensive studies have also validated the efficacy of Traditional Chinese Medicine (TCM) in hepatoprotective approaches^12,13^. Ginsenoside Rd (Rd) is an active component commonly found in *Panax ginseng* C.A, that includes ginseng and pseudo-ginseng, which has shown significant protective effects on the nervous and cardiovascular systems^14,15^.

Rd impacts several regulatory pathways of significance to autophagy. Studies have suggested that Rd can down-regulate NF-κB, resulting in the inhibition of iNOS and COX-2 levels in RAW 264.7 macrophage cells^16^. It has been found to inhibit the TGF-β/Smad pathway, reduce cellular autophagy, and thus suppress hepatic stellate cells (HSCs) activation^17^. Suppression of ferroptosis, alleviating CCl4-induced liver injury in mice through the cGAS/STING pathway^18^, has been observed. Additionally, regulating the ERRα-mediated P2 × 7r pathway can reduce both fibrogenesis and inflammation in hepatic fibrosis^19^. There is also evidence that Rd, when used in combination with Phosphoarginine, can suppress liver cancer by reducing HIF-1α through the PI3K/AKT/mTOR signaling pathway^20^. However, the potential protective effect of RD in modulation of autophagy on TAA-induced acute liver injury remains to be investigated.

Here, for the first time, an investigation on the hepatoprotective effect of Rd against acute liver injury induced by TAA in mice is presented. We assess the underlying mechanisms that are implicated in autophagy and inflammation. The experimental findings provide valuable evidence for prevention and treatment of acute liver injury.

## Materials and methods

### Regents and chemicals

TAA and Rd (purity ≥ 95.0%, HPLC) were obtained from Shanghai Yuanye Bio-Technology Co., Ltd. (Shanghai, China). Diammonium glycyrrhizinate was purchased from Zhengda Tianqing Pharmaceutical Group Co., Ltd. (Nanjing, China). Rapamycin and GSK621 were purchased from Med Chem Express Biotech Co. Ltd. (NJ, USA). Aspartate aminotransferase (AST), alanine aminotransferase (ALT), glutathione S-transferase (GSH-ST), and lactate dehydrogenase (LDH) assay kits were obtained from Nanjing Jiancheng Bioengineering Research Institute (Nanjing, China). H&E staining kit was acquired from Beijing Solarbio Science and Technology Co., Ltd. (Beijing, China). Tris-EDTA, 0.25% Triton X-100, and 10% fetal bovine serum were acquired from Sigma-Aldrich Life Science and Technology Co., Ltd. (Wuxi, China). The immunohistochemistry (IHC) staining kit was purchased from Maixin Biotechnology Co., Ltd. (Fuzhou, China). The Rabbit mAb of SQSTM1/P62, LC3II/I Beclin1, phospho ULK1, ULK1, phospho mTOR, mTOR, and NLRP3 were obtained from Cell Signaling Technology (MA, USA). Rabbit mAb of COX-2, iNOS, α-SMA, and AMPK, as well as HRP Goat Anti-Mouse IgG (H + L) and HRP Goat Anti-Rabbit IgG (H + L), were purchased from Proteintech Group, Inc. (Wuhan, China). Rabbit mAb of phospho AMPK, IL-18, and IL-1β were purchased from Abcam Ltd. (Cambridge, UK). β-actin Mouse mAb was purchased from TransGen Biotech Co., Ltd. (Beijing, China). The annexin V-FITC Apoptosis Detection Kit was purchased from Beyotime Biotechnology (Shanghai, China). Unless otherwise indicated, all other reagents and chemicals were sourced from Beijing Chemical Factory (Beijing, China).

### Animals

A total of 48 healthy specific pathogen-free (SPF) C57BL/6 mice (8 weeks), obtained from Shanghai Slac Laboratory Animal Co. Ltd. [Shanghai, China, Production License: SCXK (Zhejiang) 2019–2020], with body weight of 20 ± 2 g, were utilized in the animal experiments. The mice were accommodated at the Experimental Animal Center of Fujian University of Traditional Chinese Medicine [SYXK (Min) 2019-0007), with ad libitum access to food and water, under a 12 h light-dark cycle. All procedures were performed in accordance with the ‘Guide for the Care and Use of Laboratory Animals’ published by the National Institutes of Health, and comply with the ARRIVE guidelines. The study was approved by the ‘Animal Care and Use Committee’ of the Fujian University of Traditional Chinese Medicine, and all procedures strictly adhered to the regulations regarding animal welfare (Approval Number: FJTCM IACUC2021068).

### Animal experimental design

Forty-eight eight weeks old C57BL/6 mice were randomly assigned into the following groups (*n* = 8): control group (CON), model group (MOD), Rd low dose (12.5 mg/kg, Rd-12.5), medium dose (25 mg/kg, Rd-25), high dose (50 mg/kg, Rd-50) groups, and diammonium glycyrrhizinate group (30 mg/kg, DG). DG and Rd were suspended in 0.5% sodium carboxymethylcellulose (CMC-Na) solution. The mice were given gavage 10 mL/kg, once a day for three days continuously. Two hours after the last drug administration, TAA (50 mg/kg) dissolved in physiological saline was injected into the abdominal cavity to induce modeling. After modeling, food was restricted, but water was allowed. The mice were weighed and sacrificed by using 1.5% sodium pentobarbital (0.15 mL/10 g) for anesthesia. The livers were isolated and weighed. Liver index (%) = liver weight/body weight × 100%.

### Measurement of enzymes in serum

The blood was collected and centrifugated at 3500 rpm for 8 min to collect the upper layer of serum and stored in a − 80 °C freezer. The levels of AST, ALT, LDH, and GST in the serum were measured strictly, according to the instructions provided in the corresponding commercial kits^21^.

### Pathological staining of liver tissues

The procedure for pathological staining was conducted in accordance with our previous study^22^. Briefly, liver tissues were collected, fixed, and then washed with PBS buffer. A 0.5 cm × 0.5 cm × 0.5 cm section of liver tissue was cut and used for pathological staining. The tissues underwent dehydration using increasing ethanol concentrations, followed by rinsing with xylene I (50% ethanol, 50% xylene) and xylene II (100% xylene). Subsequently, the tissues were embedded in paraffin. The paraffin-embedded section was sliced into 4 μm thickness and dried using heat. After cleaning the paraffin section with xylene I and xylene II, it was hydrated with decreasing ethanol concentrations. Following H&E staining, the liver tissue sections were dehydrated, and pathological changes were observed under an optical microscope. Representative areas were then captured by the microscope.

### Immunohistochemistry

Following deparaffinization and hydration, the slides underwent antigen retrieval by treatment with Tris-EDTA at 100 °C for 20 min in a water bath. Subsequently, permeabilization was achieved using 0.25% Triton X-100 for 10 min. Non-specific binding sites were blocked with 10% fetal bovine serum in 1× TBS for 30 min. The tissue sections were then incubated with anti-αSMA overnight at 4 °C, followed by incubation with an anti-(HRP)-conjugated secondary antibody for 1.5 h at room temperature. Positive signals were visualized using an IHC staining kit. Quantification of the positive area was performed on ten randomly selected lobule micrographs from each slice, utilizing the green channel of RGB stack mode in ImageJ software version 1.52a.

### Cells experimental design

Since the activation of HSC is a critical pathological process during acute liver injury and serves as an important in vitro research target for liver injury. To demonstrate the impact of hepatic stellate cells on liver cell damage, we firstly treated HSC-T6 cells with LPS for 24 h, then treated AML12 cells with the conditioned medium (CM) containing 50% of culture supernatant of hepatic stellate cells and then detected the apoptosis of both cell types using flow cytometry. A rat hepatic stellate cell line (HSC-T6) and Alpha Mouse Liver 12 (AML-12) were procured from Kunming Cell Bank of Chinese Academy of Sciences in Kunming, China. The HSC-T6 cells were cultured in complete medium with 10% FBS and 1% insulin-transferrin-selenium, and maintained at a temperature of 37 °C under a 5% CO_2_ atmosphere. The AML-12 cells were cultured under humidified 5% CO_2_ at 37℃ in complete medium, which was made from 89% DMEM, 1% insulin-transferrin-selenium, 10% FBS and 40 ng/mL dexamethasone. HSC-T6 cells were treated with G-Rd at concentrations of 2.5, 5, or 10 µM, either with or without LPS stimulation at 100 ng/mL. In addition, a group of cells treated with G-Rd at 10 µM was co-incubated with the mTOR inhibitor RAPA at 2 µM, or the AMPK activator GSK621 at 10 µM. The cells were then further incubated for a period of 12 h, after which protein extraction was carried out.

### Cell viability assays

Briefly, the MTT proliferation assay was employed to assess the cell viability of HSC-T6 cells. Initially, a 96-well culture plate was prepared, with each well containing 5 × 10^4^ cells in 1 mL of culture medium. After 24 h, Rd was added to each well (100 µL of drug volume per well). Another 24 h later, 22 µL of 5 mg/mL MTT solution was added to each well, resulting in a final concentration of MTT of 0.5 mg/mL. The plate was then placed in an incubator for 4 h, followed by careful removal of the supernatant. Subsequently, 200 µL of DMSO was added to each well. The plate was placed in a microplate reader, shaken for 5–10 min to facilitate the dissolution of the formazan crystals, and the optical density (OD value) was measured at a wavelength of 490 nm.

### Annexin V/PI apoptosis staining

HSC-T6 and AML-12 cells were plated at a density of 5 × 10^4^ cells per well in six-well plates and incubated for 24 h. Subsequently, both cell lines were stimulated with 100 ng/mL of LPS for 12 h. The supernatants from HSC-T6 cells were collected and centrifuged at 10,000 rpm for 10 min, followed by mixing with complete medium in a 1:1 ratio to generate conditioned medium (CM). Cell apoptosis was assessed using an Annexin V/PI staining kit according to the manufacturer’s instructions. In brief, after harvesting and washing, the cells were resuspended in 195 µL of binding buffer and stained with 5 µL of Annexin V-FITC and 10 µL of PI in the dark for 15 min. The percentages of viable, early apoptotic, and late apoptotic cells were quantified by flow cytometry.

### Immunofluorescence staining assay

Liver tissue sections with a thickness of 5 μm were reprocessed with xylene, followed by a gradient of ethanol. Then, the sections were incubated with a normal goat serum-blocking solution by adding it dropwise and incubating at room temperature for 20 min. The LC3II primary antibody (dilution ratio of 1:70) was added dropwise and incubated at 4 °C overnight. The following day, the sections were taken out, allowed to warm up, washed once with PBS, and air-dried. Fluorescent-labeled secondary antibody (IgG) was added dropwise, incubated at 37 °C in the dark for 20 min, washed once with PBS, air-dried, and then mounted with neutral resin. Fluorescence inverted microscope (Leica, DMi8, Germany) was used for image capture and analysis.

### qPCR analysis of IL-6, TNF-α, iNOS, COX-2 mRNA expressions

The total RNA was extracted from the powdered frozen liver tissues, and the quantity and purity were assessed using a Thermo Fisher Scientific nanodrop spectrophotometer. The RT Reverse Transcription kit was used to reverse transcribe 200 ng of total RNA into cDNA according to the kit’s instructions. The protocol involved incubating the reaction mixture at 25.0 °C for 5 min, 42.0 °C for 60 min, 70.0 °C for 5 min, followed by cooling to 4.0 °C. The resulting cDNA samples were diluted with RNase-free ddH_2_O and mixed with the ChamQ SYBR qPCR Master Mix before undergoing stem-loop RT-PCR using the ABI7900 system from Applied Biosystems, a division of Thermo Fisher Scientific. Each 9 µL reaction mixture contained 4 µL of ChamQ SYBR qPCR Master Mix, 1 µL of each primer, 1 µL of ROX Reference Dye 1, 1 µL of cDNA, and 2 µL of RNase-free ddH_2_O. The primer sequences for IL-6, TNF-α, iNOS, and COX-2 are available in Table 1. The reaction conditions for the targeted genes were set as follows: initial heat activation at 95 °C for 30 s, followed by 40 cycles of denaturation at 95 °C for 10 s, annealing at 60 °C for 30 s, extension at 95 °C for 15 s and final extension at 95 °C for 15 s. Data analysis was performed using the comparative CT method (^ΔΔCT^ Method) with β-actin mRNA levels serving as the normalization control. The fold change in gene expression between the different treatments was determined.

**Table 1:** The primer sequences used in qPCR analysis.

Primers	Forward (5′→3′)	Reverse (5′→3′)
IL-6	CTGCAAGAGACTTCCATCCAG	AGTGGTATAGACAGGTCTGTTGG
TNF-α	GCCGATGGGTTGTACCTTGT	TCTTGACGGCAGAGAGGAGG
iNOS	GAAGGGGACGAACTCAGTGG	GTGGCTCCCATGTTGCATTG
COX-2	GCCTGGTCTGATGATGTATGC	CCTATGAGTATGAGTCTGCTGGTT
β-actin	TGTCCACCTTCCAGCAGATGT	AGCTCAGTAACAGTCCGCCTAG

### Western blot analysis

The total protein was extracted from each group’s liver tissues with 0.5 mL RIPA assay buffer containing 1% protease/phosphatase inhibitor cocktail. Total protein was mixed with 5× protein loading buffer at a concentration 0.5 mg/mL and denatured at 100 °C for 10 min. Subsequently, the proteins were separated by SDS-PAGE electrophoresis (PowerPac HC, BioRAD) at 90 V for 90 min using protein gels. The proteins in the gel were then transferred onto a polyvinylidene difluoride membrane and incubated with 5% skim milk dissolved in TBST for 70–90 min at room temperature. The membranes were subsequently incubated overnight at 4 °C with the following primary antibodies: anti-COX-2 (1:1000), anti-iNOS (1:1000), anti-NLRP3 (1:1000), anti-IL-18 (1:1000), anti- IL-1β (1:1000), anti-β-actin (1:7500), anti-SQSTM1/p62 (1:1000), anti-p-AMPK (1:750), anti-AMPK (1:750), anti-p-mTOR (1:1000), and anti-mTOR (1:1000), anti-LC3I/II (1:1000), anti-Beclin1 (1:1000), anti-p-ULK1 (1:1000), and anti-ULK1 (1:1000). After three washes with TBST buffer, they were co-incubated for 1 h with either anti-rabbit or anti-mouse secondary antibodies conjugated with horseradish peroxidase (1:8000). β-actin, purchased from TransGen Biotech, Beijing, China (1:2000), served as the internal control. The immunoreactive bands were visualized. Image Lab 6.0 was utilized for band intensity quantification.

### Statistical analysis

All data was analyzed using SPSS 26.0 statistical software, and the values were expressed as mean ± standard error of the mean (SEM). Statistical analysis was conducted using one-way analysis of variance (ANOVA). In cases where the data did not follow a normal distribution, the non-parametric Mann–Whitney *U* test was utilized. When the data followed a normal distribution and exhibited equal variances, the LSD test was used. In instances of unequal variances, the Games-Howell test was employed for statistical analysis. Statistical significance was considered at values of *p* < 0.05.

## Results

### Rd exhibited protection against TAA-induced acute liver injury

In this study, a commonly used animal model of acute liver injury was established through the injection of TAA^23^. This model is characterized by heightened levels of ALT, AST, GST, LDH in the serum^24^, and increased liver index^25^, along with histological changes, pathological alterations and HSC activation in liver tissue.

**Fig. 1:**
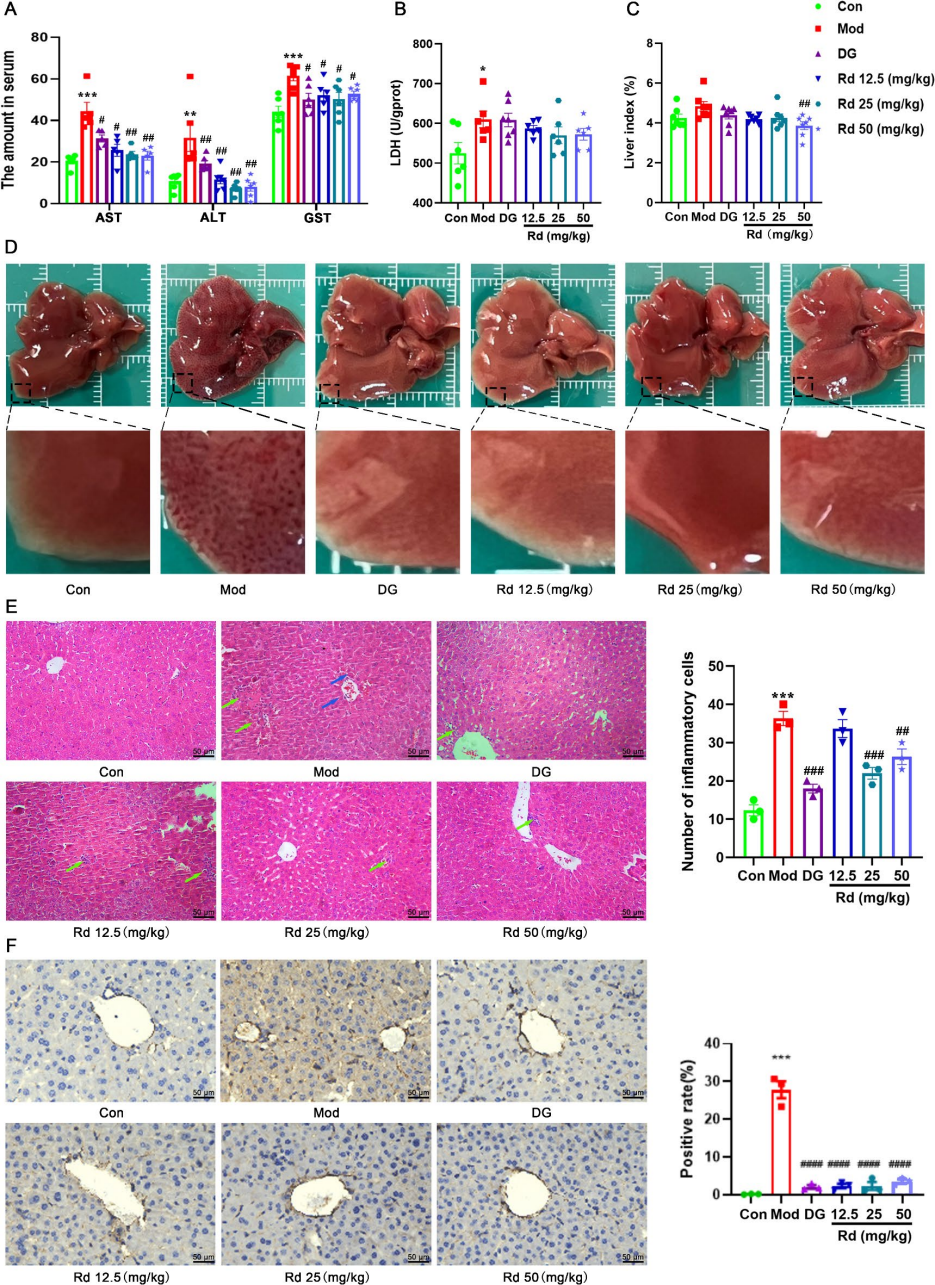
Rd ameliorated TAA-induced acute liver injury in mice. (**A**,**B**) AST, ALT, GST and LDH levels in serum of liver-injured mice (*n* = 6). (**C**) Liver index in mice with acute liver injury (*n* = 6). (**D**) Effect of Rd on the appearance of liver tissue in mice (*n* = 6). (**E**) Pathological changes and number of inflammatory cells in mouse liver histological Sect. (200×, *n* = 3). Scale bar, 50 μm. Red arrows indicated inflammatory cell infiltration. The blue arrow indicates nuclear condensation. (**F**) Expression and quantification of α-SMA in immunohistochemical micrograph (200×, *n* = 3). Scale bar, 50 μm. Values are shown as the mean ± SEM; *p < 0.05, **p < 0.01, ***p < 0.001 vs. control group, ^#^*p* < 0.05, ^##^*p* < 0.01, ^###^*p* < 0.001 vs. model group, analyzed by one-way ANOVA with Dunnett’s test.

To investigate whether Rd had hepatoprotective effects, the TAA-induced acute liver injury model in mice were pre-treated with Rd. No mortality was observed among the experimental mice in each group. Compared with the control group, the model group showed significant increase in serum levels of AST (*p* < 0.001), ALT (*p* < 0.01), GST (*p* < 0.001), and LDH (*p* < 0.05). All mice in the DG (30 mg/kg) and Rd groups showed a noteworthy reduction in the levels of AST, ALT, and GST (*p* < 0.05 or *p* < 0.01) compared to the model group (Fig. 1A). Meanwhile, there was a measurable decline of LDH in Rd 50 mg/kg group (Fig. 1B). As observed in Fig. 1C, the high-dose group of Rd exhibited a significant reduction in liver index compared to the model group.

Morphologic observation of the liver revealed a smooth surface with normal color in the control group, while the model group exhibited numerous dark red spots on the liver surface. Comparatively, the DG and Rd groups showed varying degrees of improvement, with the medium dose Rd (25 mg/kg) group exhibiting the most prominent effect (Fig. 1D). To assess whether Rd leads to pathological changes in liver tissue, pathological staining was performed. Histopathological analysis of the control group using H&E staining showed, as expected, well-organized liver cells with normal hepatic cord morphology and intact hepatic lobule structure. In contrast, the model group displayed nuclear pyknosis, abnormal hepatocyte arrangement, tissue infiltration of inflammatory cells, and areas of hemorrhage. Intervention with Rd and DG ameliorated these pathological changes (Fig. 1E), resulting in restoration of normal hepatic lobule structure, alleviated infiltration of inflammatory cells.

Hepatic inflammatory responses are frequently correlated with the activation of HSC, which undergo a transition from a quiescent to an activated state upon liver injury^26^. This phenomenon was substantiated by the elevated expression of the HSC activation marker α-SMA (Fig. 1F) in the Mod groups. Conversely, the levels observed in the DG and RD groups were comparable to those in the control group, exhibiting low expression levels.

### Rd inhibited inflammatory response in acute liver injury

**Fig. 2:**
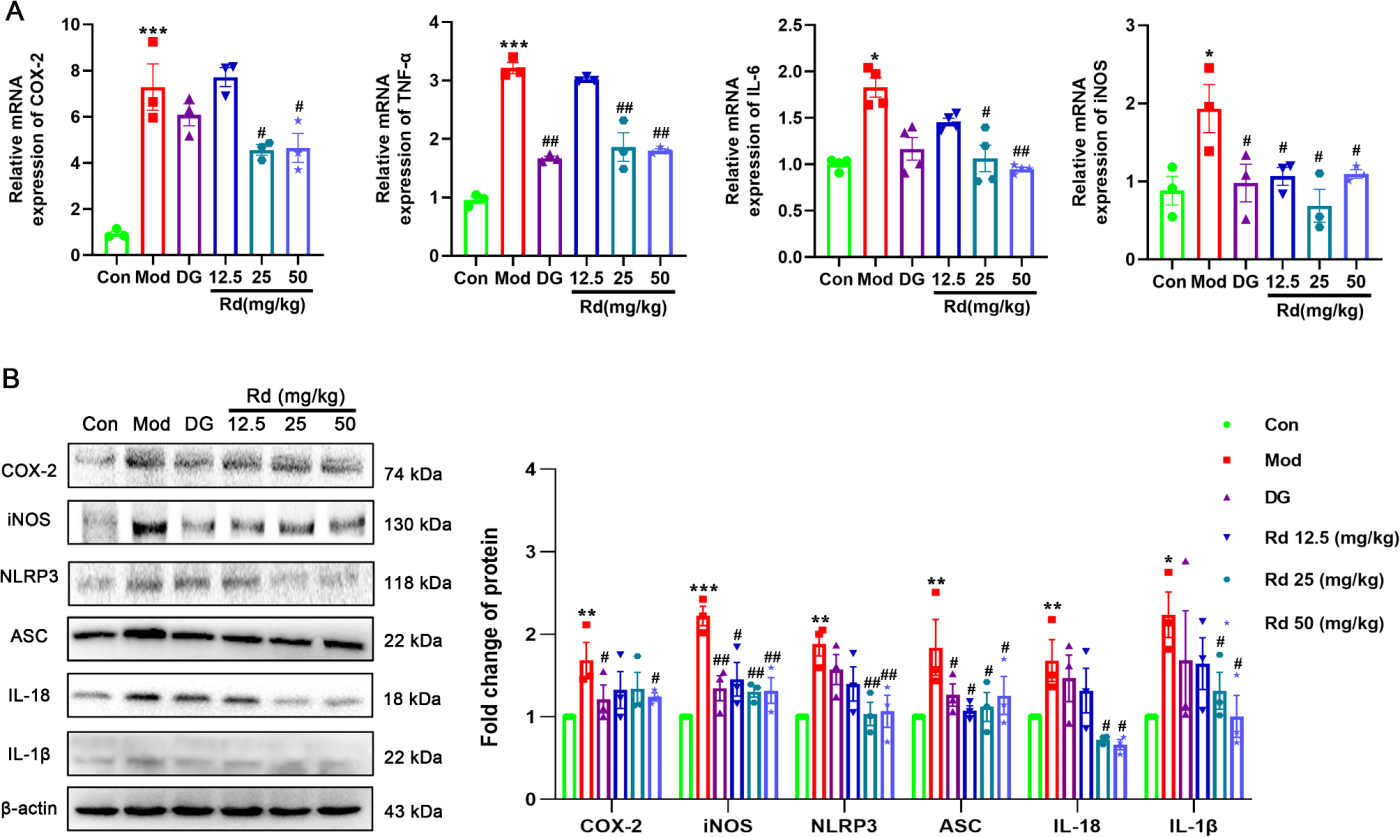
Rd alleviated hepatic tissue inflammation in mice with acute liver injury. (**A**) COX-2, TNF-α, IL-6, iNOS, mRNA expression in liver (*n* = 3). (**B**) COX-2, iNOS, NLRP3, ASC, IL-18, IL-1β protein expression in liver (*n* = 3). Values are shown as the mean ± SEM; *p < 0.05, **p < 0.01, ***p < 0.001 vs. control group, ^#^*p* < 0.05, ^##^*p* < 0.01 vs. model group.

During the development of acute liver injury, various pathological mechanisms are involved, with inflammation-induced hepatocellular damage being one of the key mechanisms. Herein, after injury, the qPCR results revealed a significant increase (*p* < 0.05 or *p* < 0.001) in the mRNA expression of pro-inflammatory cytokines (COX-2, TNF-α, IL-6, and iNOS) in the model group. After low dose of Rd (12.5 mg/kg) and DG treatments, there was no significant effect on the mRNA expression of COX-2 and IL-6. However, the medium and high doses (25, 50 mg/kg) of Rd demonstrated a significant downregulation effect on their expression (*p* < 0.05 or *p* < 0.01) (Fig. 2A).

Concomitantly, after injury Western blot analysis revealed a significant upregulation (*p* < 0.05, or *p* < 0.01, or *p* < 0.001) in the protein expression levels of inflammatory markers COX-2, iNOS NLRP3, ASC, IL-18 and IL-1β, in the liver tissue of the model group compared to the control group. However, after intervention with DG and Rd, there was a notable reduction (*p* < 0.05 or *p* < 0.01) observed in these markers (Fig. 2B).

### Rd regulated autophagy in acute liver injury via inhibiting the AMPK/mTOR/ULK1 axis

**Fig. 3:**
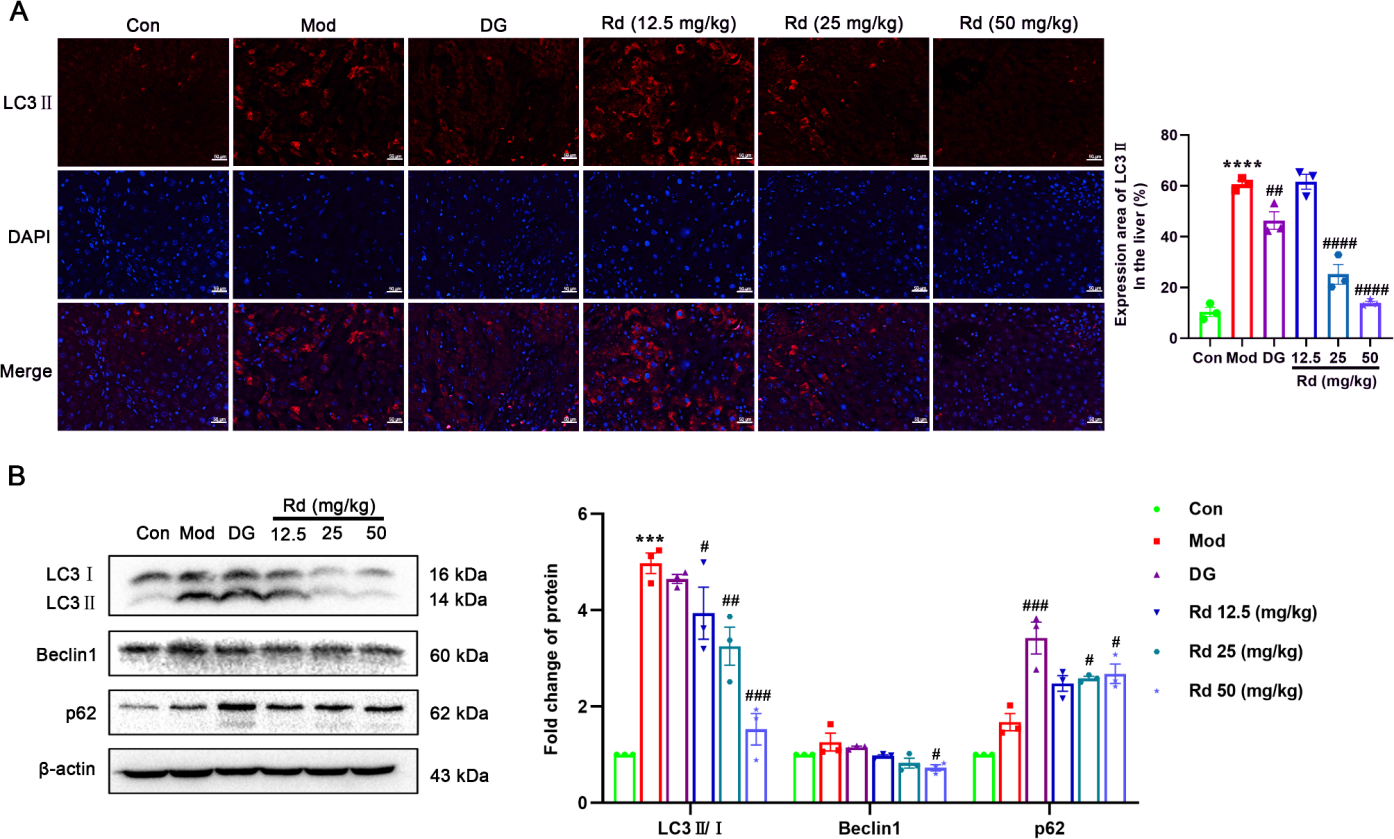
Rd reduced hepatic tissue autophagy levels in mice with acute liver injury. (**A**) LC3II protein expression in liver tissues (400×, *n* = 3). (**B**) LC3, Beclin1, p62 protein expression in liver (*n* = 3). Values are shown as the mean ± SEM, ***p < 0.001, ****p < 0.0001 vs. control group, ^#^*p* < 0.05, ^##^*p* < 0.01, ^###^*p* < 0.001, ^####^*p* < 0.0001 vs. model group.

Studies have shown that regulating autophagy can alleviate inflammation and stress levels to improve acute liver injury^27,28^. To investigate the impact of Rd on autophagy in this ALI model, the expression levels of autophagy-related proteins and upstream signaling pathways were examined. Immunofluorescence analysis revealed a significant elevation (*p* < 0.001) in both the area and intensity of LC3II in the liver tissue of the model group. In the DG group, there was a partial decrease of fluorescence intensity and area (Fig. 3A). Notably, Rd demonstrated a significant decrease in both the fluorescence area and intensity of LC3II (*p* < 0.001).

Western blot results showed that significant higher levels of autophagy-associated proteins LC3II/I in the model group compared to the control group (*p* < 0.001), and a noticeable increase in the autophagy substrate p62. Compared with the model group, the levels of Beclin1 and LC3II/I were considerably decreased, and the expression level of p62 was further increased after Rd treatment (*p* < 0.05, *p* < 0.01, or *p* < 0.001) (Fig. 3B).

**Fig. 4:**
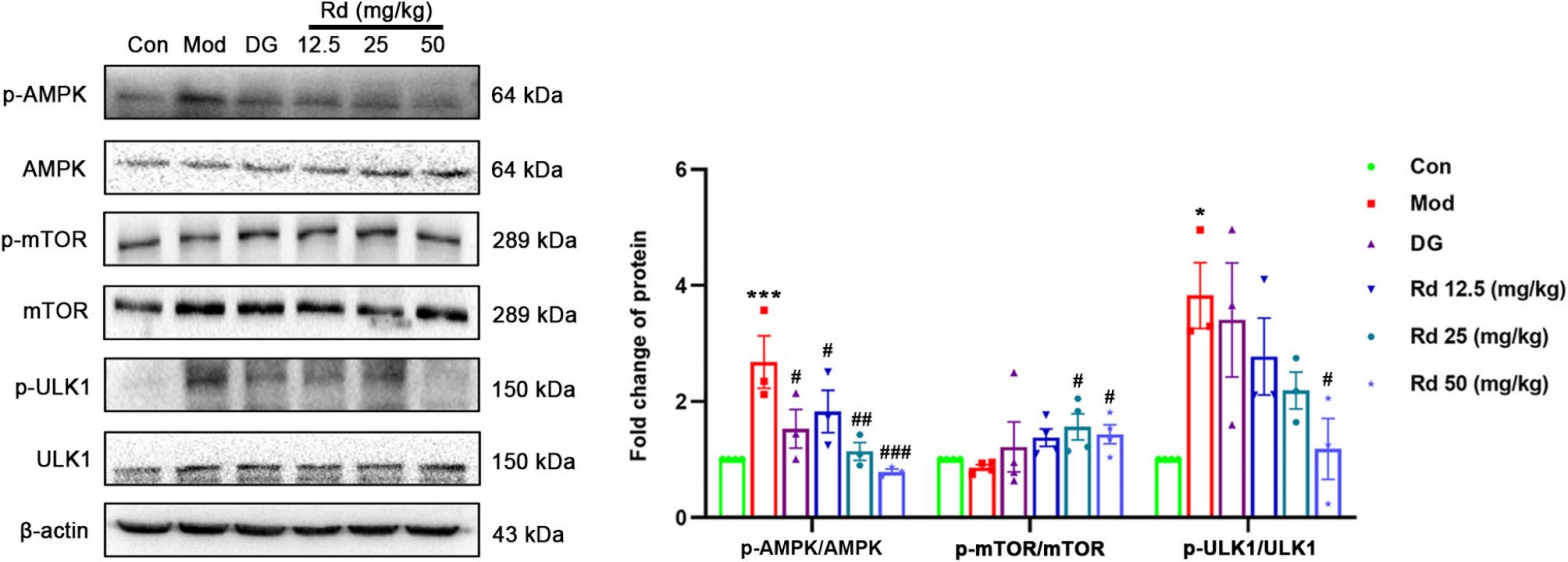
Rd regulated AMPK pathway in mice liver with acute liver injury. AMPK, mTOR, ULK1 phosphorylation levels in mice liver (*n* = 3). Values are shown as the mean ± SEM; *p < 0.05, ***p < 0.001, vs. control group, ^#^*p* < 0.05, ^##^*p* < 0.01, ^###^*p* < 0.001, vs. model group.

A significant increase in the phosphorylation levels of AMPK and ULK1 (*p* < 0.05 or *p* < 0.001) were also observed in hepatic tissue, along with the decreased phosphorylation levels of mTOR in the model group compared to the control group. Concomitantly, treatment with DG and Rd led to a reduction in the phosphorylation levels of AMPK and ULK1 (*p* < 0.05), and the upregulation of phosphorylation levels of mTOR (*p* < 0.05) (Fig. 4).

### Rd inhibited LPS-induced inflammation and autophagy in HSC-T6 cells

**Fig. 5:**
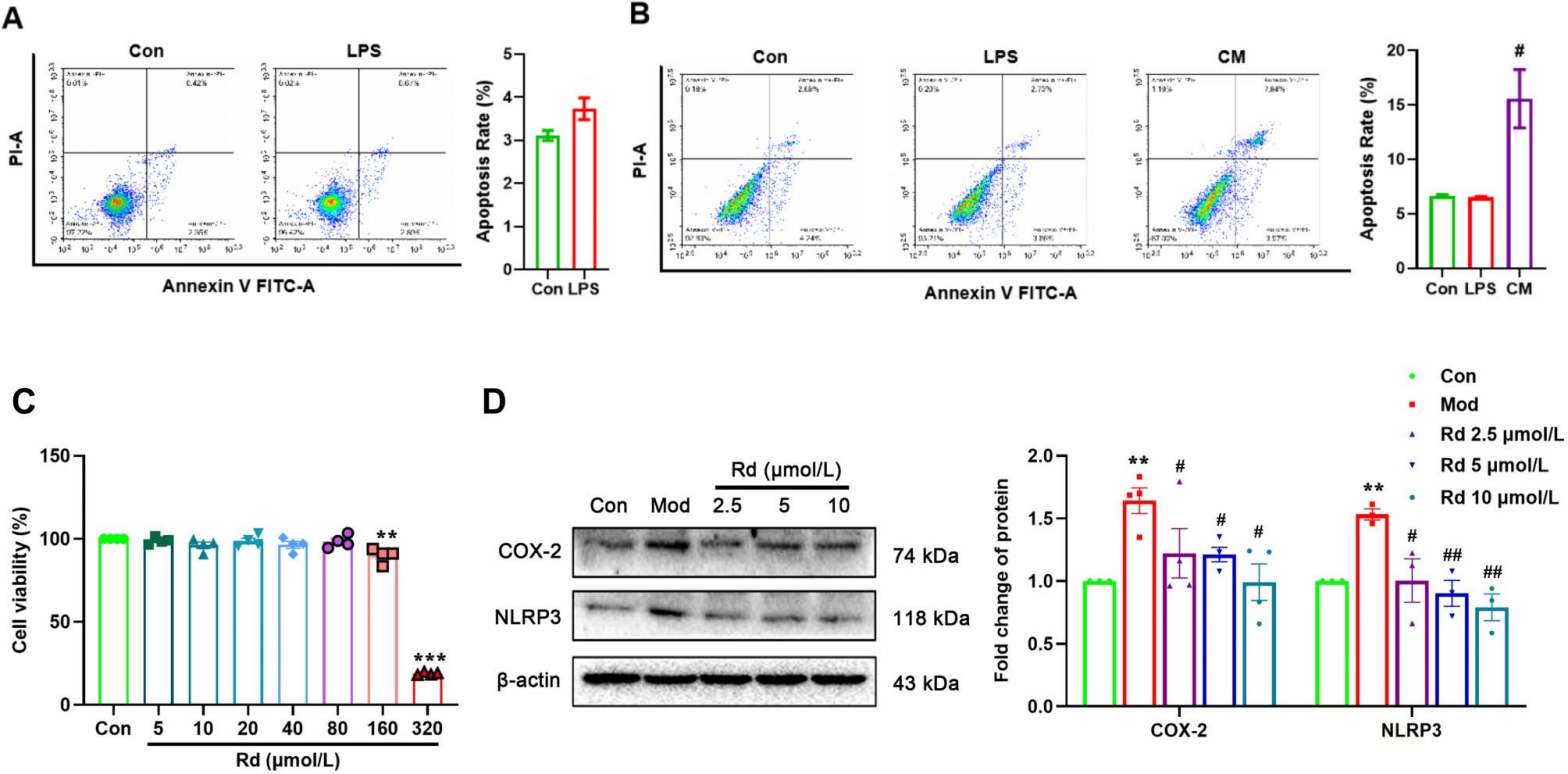
Apoptosis in HSC-T6 and AML12 cells and Rd inhibited inflammation in HSC-T6 cells. (**A**) Apoptosis of HSC-T6 cells were determined by flow cytometry after Annexin V-FITC and PI double staining, (*n* = 3). (**B**) Apoptosis of AML-12 cells, (*n* = 3), ^#^*p* < 0.05, vs. LPS group. (**C**) Viability of HSC-T6 cells after 24 h incubation with different concentrations of Rd (*n* = 4). (**D**) LPS-induced inflammation-associated protein expression in HSC-T6 cells (*n* = 3). Values are shown as the mean ± SEM, **p < 0.01, ***p < 0.001 vs. control group, ^#^*p* < 0.05, ^##^*p* < 0.01 vs. model group.

In the liver tissue, hepatocytes cells constitute the major cell population, accounting for 60–80% of the total cell number^29^. During acute liver injury, these hepatocytes cells undergo damage, concurrently with the activation of hepatic stellate cells. Our results demonstrated that LPS treatment at a concentration of 100 ng/mL did not significantly induce apoptosis in either HSC-T6 cells or AML12. However, when AML12 cells were treated with conditional medium derived from HSC-T6 cells for 12 h, a significant increase in apoptotic cells were observed compared to the control group (Fig. 5A, B). Therefore, it is suggested that the activation of HSC-T6 cells plays an important role in acute liver injury, so we focused on the effects of Rd on HSC-T6 cells in the following in vitro experiments.

Firstly, we evaluated the concentration of Rd affecting cell viability. Results from an MTT assay showed that after 24 h of incubation, Rd did not significantly affect cell viability up to a concentration of 160 µM (*p* < 0.01). A significant effect was observed at 320 µM (*p* < 0.001). Therefore, the selected concentrations of Rd (2.5–10 µM) in our experiments did not exhibit significant toxicity to HSC-T6 cells (Fig. 5C).

After the activation of HSC-T6 by LPS, the expression levels of NLRP3 and COX-2 were significantly increased compared to the control group (*p* < 0.01). However, following intervention with Rd, the expression levels of NLRP3 and COX-2 were significantly decreased compared to the model group (*p* < 0.05 or *p* < 0.01) (Fig. 5D).

Numerous studies have demonstrated that the activation of HSC-T6 cells is accompanied by an increase in autophagy levels^30^, which can be blocked by autophagy inhibitors.

**Fig. 6:**
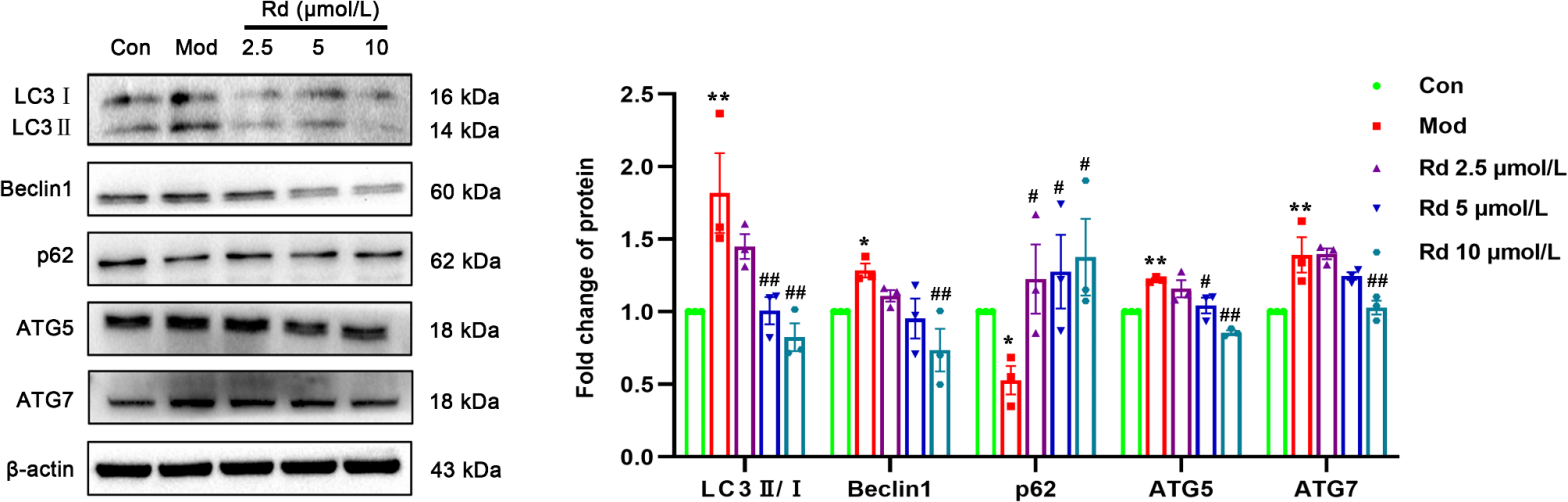
Rd inhibited LPS-induced autophagy in HSC-T6 cells. LPS-induced autophagy-related protein expression in HSC-T6 cells (*n* = 3). Values are shown as the mean ± SEM; *p < 0.05, **p < 0.01, vs. control group, ^#^*p* < 0.05, ^##^*p* < 0.01 vs. model group.

The expression levels of autophagy-related genes showed significant changes, as evidenced by the data presented in Fig. 6. Compared with the control group, the expressions of autophagy-related gene 5 (ATG5) and autophagy-related gene 7 (ATG7) were significantly increased (*p* < 0.01). Concomitantly, the levels of Beclin1 and LC3II/I were also significantly increased (*p* < 0.05 or *p* < 0.01), and the expression of p62 decreased (*p* < 0.05). After intervention with Rd, the levels of ATG5, ATG7, LC3II/I and Beclin1 decreased significantly (*p* < 0.05 or *p* < 0.01), while the expression of p62 increased (*p* < 0.05).

**Fig. 7:**
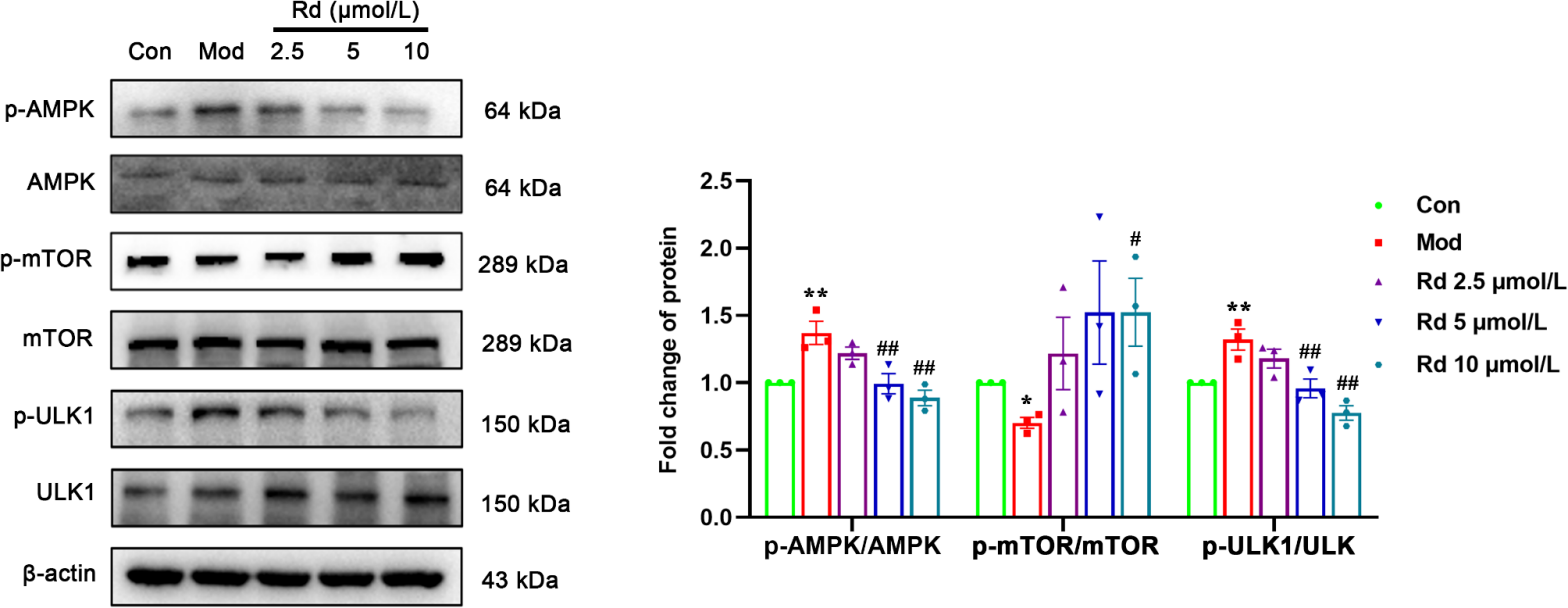
Rd regulated AMPK pathway in LPS-induced HSC-T6 cells. AMPK/mTOR/ULK1 pathway in HSC-T6 cells (*n* = 3). Values are shown as the mean ± SEM; *p < 0.05, **p < 0.01, vs. control group, ^#^*p* < 0.05, ^##^*p* < 0.01 vs. model group.

To assess potential regulatory effects of Rd on the AMPK/mTOR/ULK1 pathway in HSC-T6 cells, Western blot experiments were performed. In comparison to the control group, LPS substantially increased the phosphorylation levels of p-AMPK and p-ULK1 proteins (*p* < 0.01), while decreasing the phosphorylation level of p-mTOR (*p* < 0.05). Following Rd intervention, as compared to the model group, there was a decrease in the phosphorylation levels of AMPK and ULK1 (*p* < 0.01) and an increase in the phosphorylation level of mTOR (*p* < 0.05) (Fig. 7).

### Rapamycin and GSK621 reversed the inhibitory effect of Rd on inflammation and autophagy in HSC-T6 cells

**Fig. 8:**
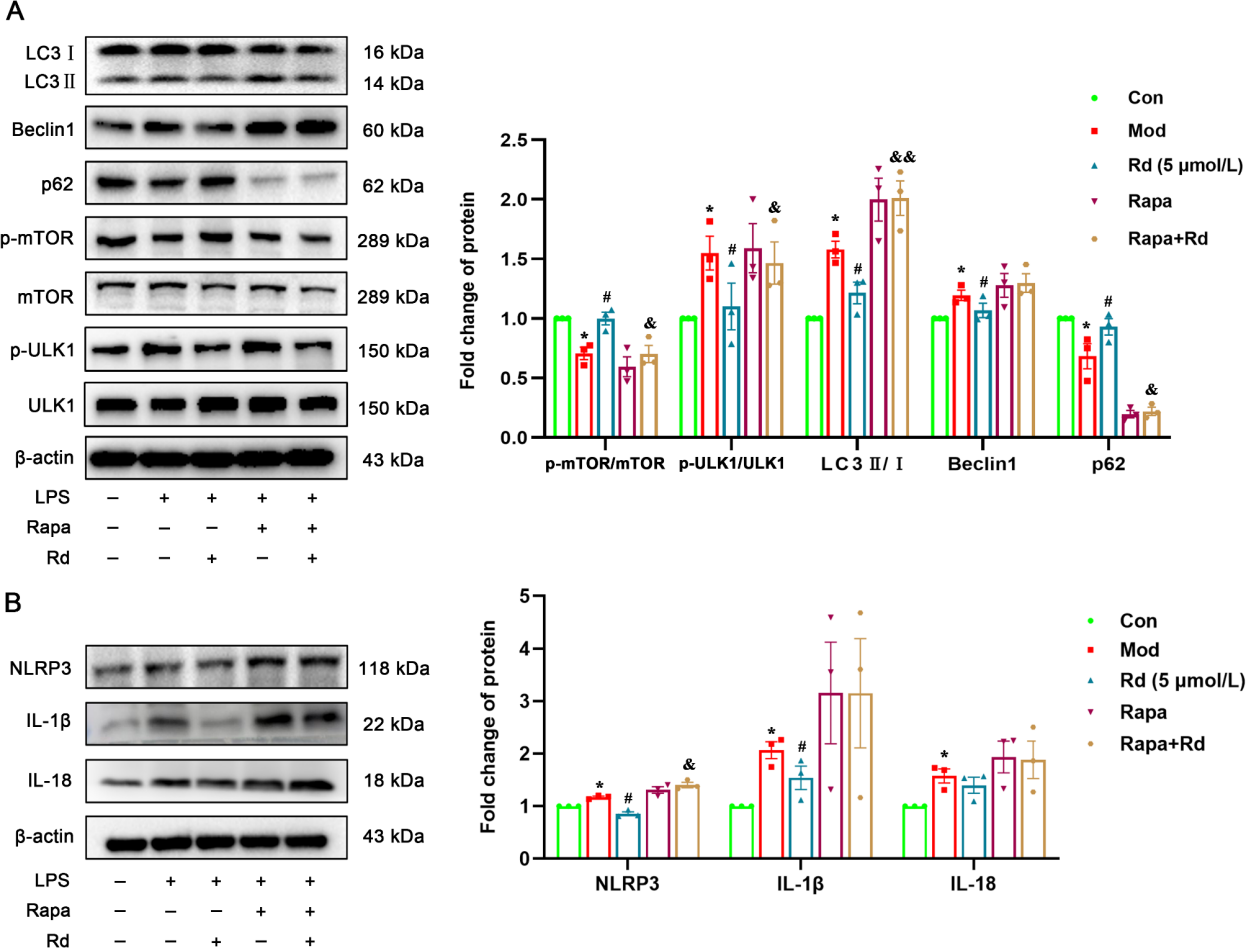
Rapamycin reversed the inhibitory effect of Rd on inflammation and autophagy in HSC-T6 cells. (**A**) Autophagy-associated protein in HSC-T6 cells (*n* = 3). (**B**) Inflammation-associated protein expression (*n* = 3). Values are shown as the mean ± SEM; *p < 0.05 vs. control group, ^#^*p* < 0.05 vs. model group, ^&^*p* < 0.05, ^&&^*p* < 0.01 vs. treatment group.

The results from the previous section of in vitro experiments demonstrated that Rd regulated autophagy through the AMPK/mTOR/ULK1 signaling pathway and reduced the expression of the inflammasome NLRP3. To verify whether modulating autophagy can improve the expression of the inflammasome, mTOR inhibitor rapamycin and AMPK activator GSK621 were employed to induce high levels of autophagy in HSC-T6 cells. Following rapamycin intervention, the LPS + Rapamycin treatment showed a significant increase in the LC3 II/I ratio compared to the model group. This was accompanied by elevated expression of Beclin1 and phosphorylation level of ULK1. Concomitantly, a notable decrease in p62 expression was observed. Conversely, the phosphorylation level of mTOR decreased. In comparison to the LPS + Rd group, the LPS + Rapamycin + Rd group demonstrated a significant increase in the LC3 II/I ratio (*p* < 0.001). Additionally, increases in the expression of beclin1 and phosphorylation of ULK1 were observed. Concurrently, both p62 expression and the phosphorylation level of mTOR did significantly decrease (*p* < 0.05) (Fig. 8A). Furthermore, after rapamycin intervention, compared to the model group there was a significant upregulation in the expression of NLRP3, along with its downstream effectors IL-18 and IL-1β. However, the efficacy of Rd was partially attenuated under these conditions (*p* < 0.05) (Fig. 8B).

**Fig. 9:**
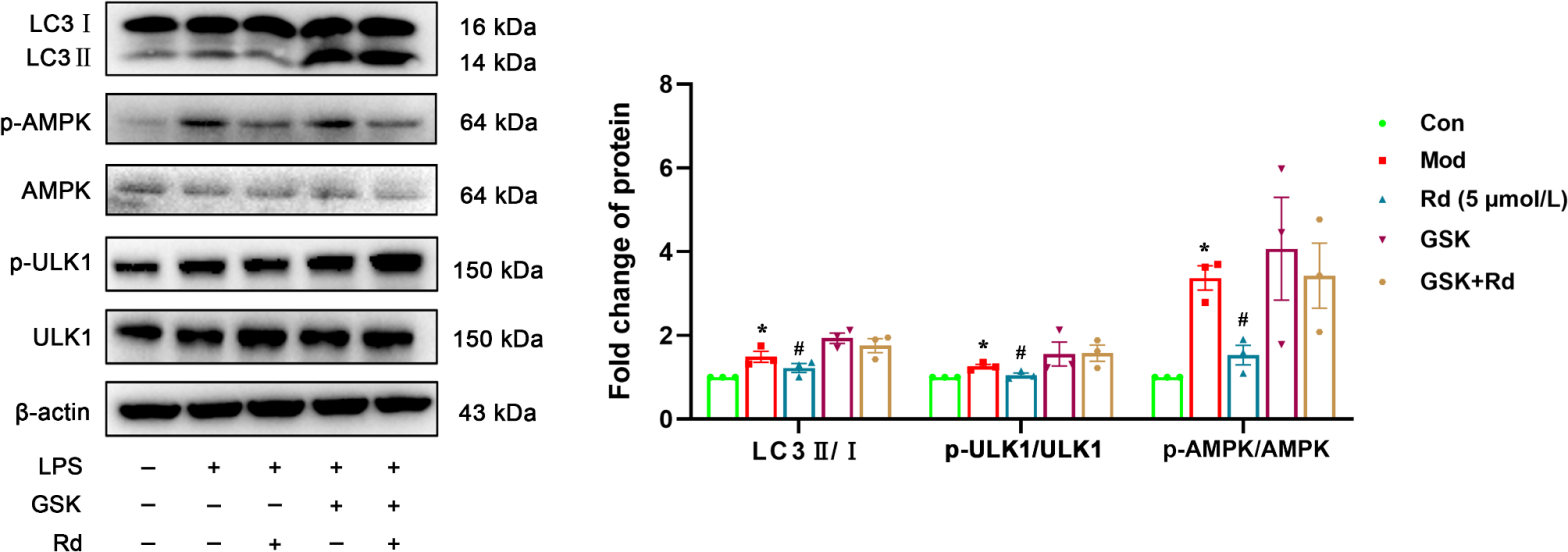
GSK621 reversed the inhibitory effect of Rd on autophagy in HSC-T6 cells. Autophagy-associated protein in HSC-T6 cells (*n* = 3). Values are shown as the mean ± SEM; *p < 0.05 vs. control group, ^#^*p* < 0.05 vs. model group.

After the intervention with GSK621, there was a significant increase in the LC3 II/I ratio compared to the model group, along with elevated phosphorylation levels of AMPK and ULK1. Similarly, the LPS + GSK621 + Rd group exhibited a comparable increase compared to the treatment group (Fig. 9).

## Discussion

TAA, known as a hepatotoxic compound, generates TAA-S-oxide and TAA-S-dioxide, leading to oxidative stress via lipid peroxidation in liver cell membranes through its intrahepatic bioactivation^31^. This oxidative stress disrupts protein synthesis, along with RNA and DNA integrity, and affects glutamyl transpeptidase activity^32,33^, which is why TAA is commonly utilized to induce hepatotoxicity in animal models. Our studies leveraging this model have uncovered that Rd pre-treatment not only mitigates inflammatory processes but also suppresses hepatic autophagy, thereby safeguarding liver tissues. Furthermore, our findings suggest a critical involvement of the AMPK/mTOR/ULK1 signaling pathway in the hepatoprotective influence exerted by Rd.

Ginsenoside Rd (C_48_H_82_O_19_), a prominent saponin derived from the root of *Panax ginseng* and *Panax notoginseng*, has diverse pharmacological effects recognized in TCM. Numerous studies have highlighted the significant protective effects of this compound in various systems, including the nervous system^15^, cardiovascular systems^14^, among others. However, the exploration of Rd’s efficacy in mitigating hepatic injury remains relatively uncharted. Our research demonstrated significant hepatoprotective effects of Rd, as evidenced by the attenuation of morphological abnormalities, a decrease in inflammatory cell infiltration, and the inhibition of HSC activation within the liver tissue. Additionally, a significant diminution in plasma aspartate AST, ALT, and GST levels was observed, corroborating the hepatoprotective capacity of Rd. These findings underscore the potential of Rd as a therapeutic agent for acute liver injury treatment^27^. Nevertheless, comprehensive studies are warranted to confirm its safety profile and therapeutic efficacy in patients with hepatic injuries.

In our study, following TAA-induced acute liver injury, analyses of both mRNA and protein expression confirm the pathogenic mechanism. It was shown that TAA-induced acute liver injury increased pro-inflammatory cytokine mRNAs IL-6, iNOS, TNF-α, and COX-2 mRNA^22^, as well as increased inflammation-related protein expression levels of COX-2, iNOS, NLRP3, IL-18, and IL-1β. In the TAA-induced acute liver injury model, endotoxemia upregulates iNOS and elevates NO levels, leading to microthrombosis and hepatocellular necrosis^34^. IL-6 is a key pro-inflammatory cytokine^35^, and NLRP3 forms an inflammasome complex that regulates inflammation^36,37^. TNF-α activates NLRP3 transcription, linked to liver diseases^3,38,39^, and downstream IL-18 and IL-1β changes^40^. Our research demonstrated that treatment with Rd reduced the expression of these pro-inflammatory cytokine mRNAs, indicating its role in lowering liver tissue inflammation. Furthermore, it also decreased the expression of inflammation-related proteins, suggesting an effect at the protein expression level rather than just gene transcription. These findings indicate that the anti-inflammatory activities of Rd in mitigating liver injury may be attributed to its ability to suppress NLRP3 inflammasome activation. This mechanism could potentially help prevent the exacerbation of liver injury.

Adequate regulation of autophagy is acknowledged as a key therapeutic target in treating liver injury^30^, and our study extends this understanding by evaluating the potential of Rd to improve liver injury through its autophagy-modulating effects. Studies have revealed the crucial roles of autophagy and inflammasomes in maintaining cellular homeostasis and managing inflammation^41^. Indeed, regulating autophagy can downregulate NLRP3 expression^8,42^ and affect IL-18 and IL-1β levels, mitigating inflammation^43^. Previous results indicated that autophagy levels were elevated in the TAA-induced model^9^, warranting further investigation of the Rd mechanism of action. This can be accomplished since sustained TAA exposure can drive liver injury to fibrosis^44^. Liver tissue experiences abnormal autophagy under TAA-induced stress, similar to AMPK/mTOR/ULK1 changes in other stress-related diseases^45,46^. These have specific roles: AMPK, is an energy sensor that regulates cellular energy metabolism, mTOR controls autophagy through mTORC1 and mTORC2 complexes^45,46^, and ULK1 initiates autophagosome formation by binding to autophagy-associated genes^47,48^. Our results indicate that Rd regulates autophagy by inhibiting AMPK phosphorylation and restoring mTOR phosphorylation.

Autophagosome regulation was evaluated through the autophagic substrate p62. During autophagosome formation, LC3I converts to LC3II, Beclin1 increases, and the autophagic substrate p62 decreases. Notably, in this study’s injured liver tissue, p62 expression slightly increased, likely due to stress responses and oxidative stress. The impact of Rd on autophagy leads to increased p62 accumulation, aligning with the observations from certain TAA-induced models^20,49^. This indicates that, consistent with previous research findings^30,50,51^, Rd alleviates inflammation and provides liver protection through autophagy regulation.

Our results thus far demonstrate that the activation of HSC-T6 cells plays a significant role in acute liver injury. We therefore assessed the effects of Rd on HSC-T6 cells in the following in vitro experiments. The effect of Rd on autophagy and pro-inflammatory factors was also investigated from response of HSCs to liver damage. In response to liver damage, HSCs undergo activation and increased autophagy due to cytokine signals^8,52^. This heightened autophagy leads to the degradation of lipid droplets within HSCs, resulting in autolysosome formation and increased pro-inflammatory cytokines IL-1β and TNF-α^4,53,54^. LPS-induced HSC-T6 activation was used to investigate the effect of Rd on autophagy and pro-inflammatory factors in this study. After HSC activation, protein expression of LC3II/I, Beclin1, ATG5, and ATG7 increased in the model group, while p62 decreased. COX-2 and NLRP3 levels also rose. Rd downregulated LC3II/I, Beclin1, ATG5, and ATG7, and upregulated p62, suggesting its autophagy regulation in vitro. Following LPS-induced activation, AMPK and ULK1 phosphorylation increased, and mTOR phosphorylation decreased in the model group. Rd reversed these changes significantly, suggesting Rd regulates HSC-T6 autophagy through the AMPK/mTOR/ULK1 pathway, which corroborates findings from recent studies^8,55^.

Diving into the cellular drama of liver pathology, we turned the spotlight on the NLRP3 inflammasome’s role within HSCs. NLRP3 inflammasome activation in HSCs is significant in liver diseases^56^. Indeed, studies have shown that regulating autophagy can inhibit NLRP3 activation and subsequently improve inflammation^57^. This study found that inducing or enhancing autophagy with LPS or rapamycin (mTOR inhibitor) led to increased NLRP3 and downstream IL-18 and IL-1β, mirroring autophagy changes. Thus, Rd regulated autophagy, inhibiting NLRP3 inflammasome activation. Indeed, overactive autophagy can exacerbate inflammation, as seen in atherosclerosis, chronic obstructive pulmonary disease, and tracheal epithelium damage^58–60^. This effect was blocked by rapamycin, revealing Rd’s anti-inflammatory mechanism by curbing excessive autophagy.

Our results opened avenues for investigating how Rd exerts anti-inflammatory actions via autophagy modulation. Future studies will focus on delineating the molecular crosstalk between autophagy-related pathways and NLRP3 inflammasome activation, aiming to identify potential biomarkers for disease progression and therapeutic response. Moreover, assessing the dose-response of Rd and similar autophagy regulators might reveal optimal therapeutic windows for liver diseases, leading to more efficacious and safer treatments.

## Conclusion

This study demonstrates that Rd exhibits a significant hepatoprotective effect against acute liver injury. This effect was manifested through a reduction in liver index, improved histopathology, and a decrease in serum markers of liver damage. We show that the hepatoprotective effect could be attributed to the downregulation of pro-inflammatory factors, suggesting the ability of Rd to alleviate acute liver injury by suppressing inflammation. Furthermore, this effect is closely associated with the regulation of autophagy. Specifically, Rd regulates autophagy through the AMPK/mTOR/ULK1 signaling pathway, both in TAA-induced acute liver injury in vivo and LPS-induced HSC-T6 cells in vitro. By inhibiting autophagy and attenuating the inflammatory response, Rd effectively mitigates acute liver injury induced by TAA and suppresses inflammation in LPS-induced HSC-T6 cells. These findings highlight the therapeutic potential of Rd in treating acute liver injury by targeting autophagy-mediated inflammation via the AMPK/mTOR/ULK1 signaling pathway.

## Electronic supplementary material

Below is the link to the electronic supplementary material.

Supplementary Material 1

Supplementary Material 2

Supplementary Material 3

## Data Availability

The data presented in this study are available on request from the corresponding author.
