# Supplementary material for: Ginsenoside Rd protects against acute liver injury by regulating the autophagy NLRP3 inflammasome pathway
Source: Sci Rep. 2025 Jan 28;15:3569. doi: 10.1038/s41598-025-87991-9 (PMC11775168; doi:10.1038/s41598-025-87991-9)
Supplement: Supplementary file 1 — Supplementary Material 1 [file 41598_2025_87991_MOESM1_ESM.pptx]

## Slide 1
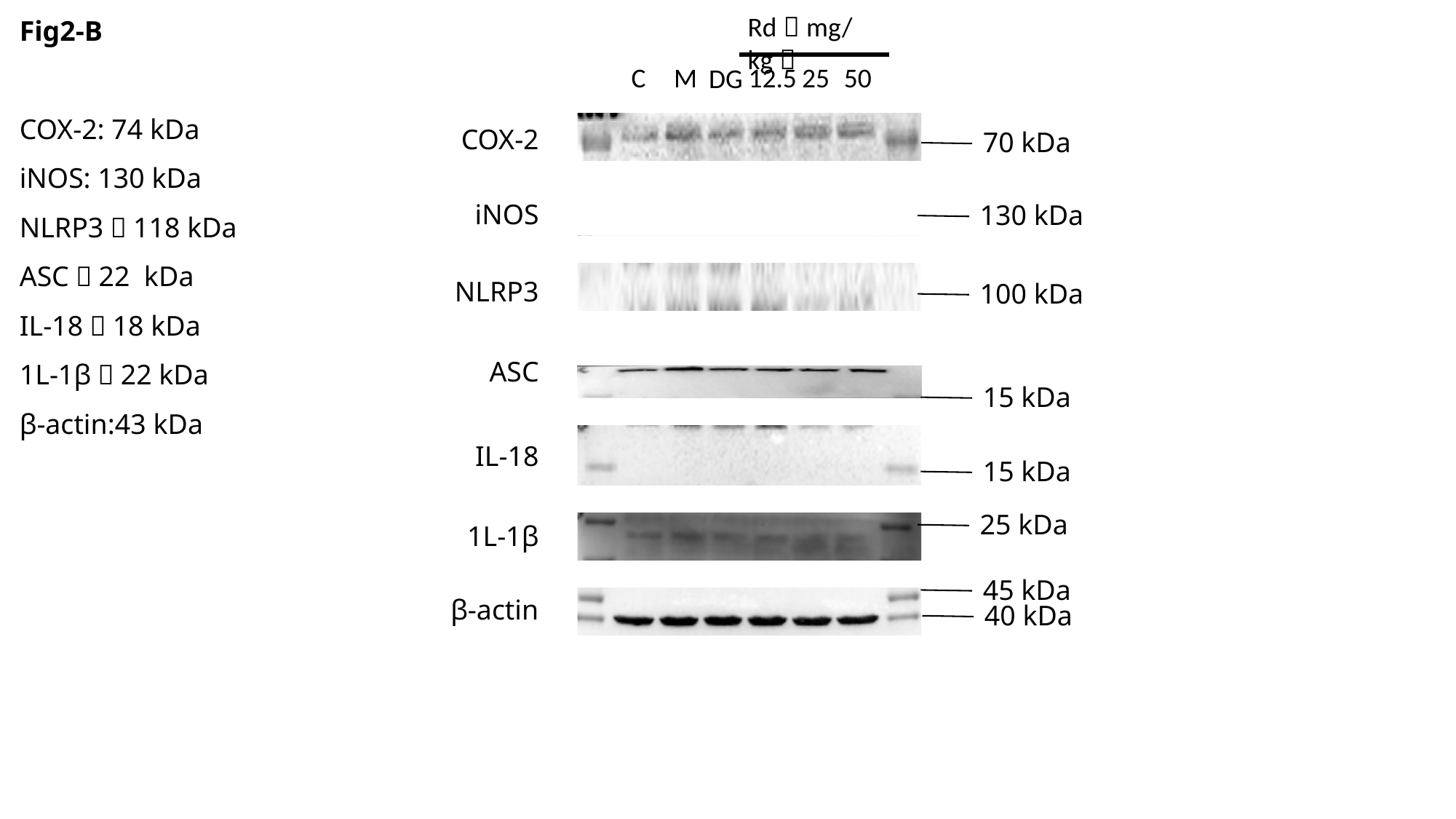

Rd（mg/kg）
12.5
25
50
C
M
DG
Fig2-B
COX-2: 74 kDa
iNOS: 130 kDa
NLRP3：118 kDa
ASC：22 kDa
IL-18：18 kDa
1L-1β：22 kDa
β-actin:43 kDa
COX-2
70 kDa
iNOS
130 kDa
NLRP3
100 kDa
ASC
15 kDa
IL-18
15 kDa
25 kDa
1L-1β
45 kDa
β-actin
40 kDa

## Slide 2
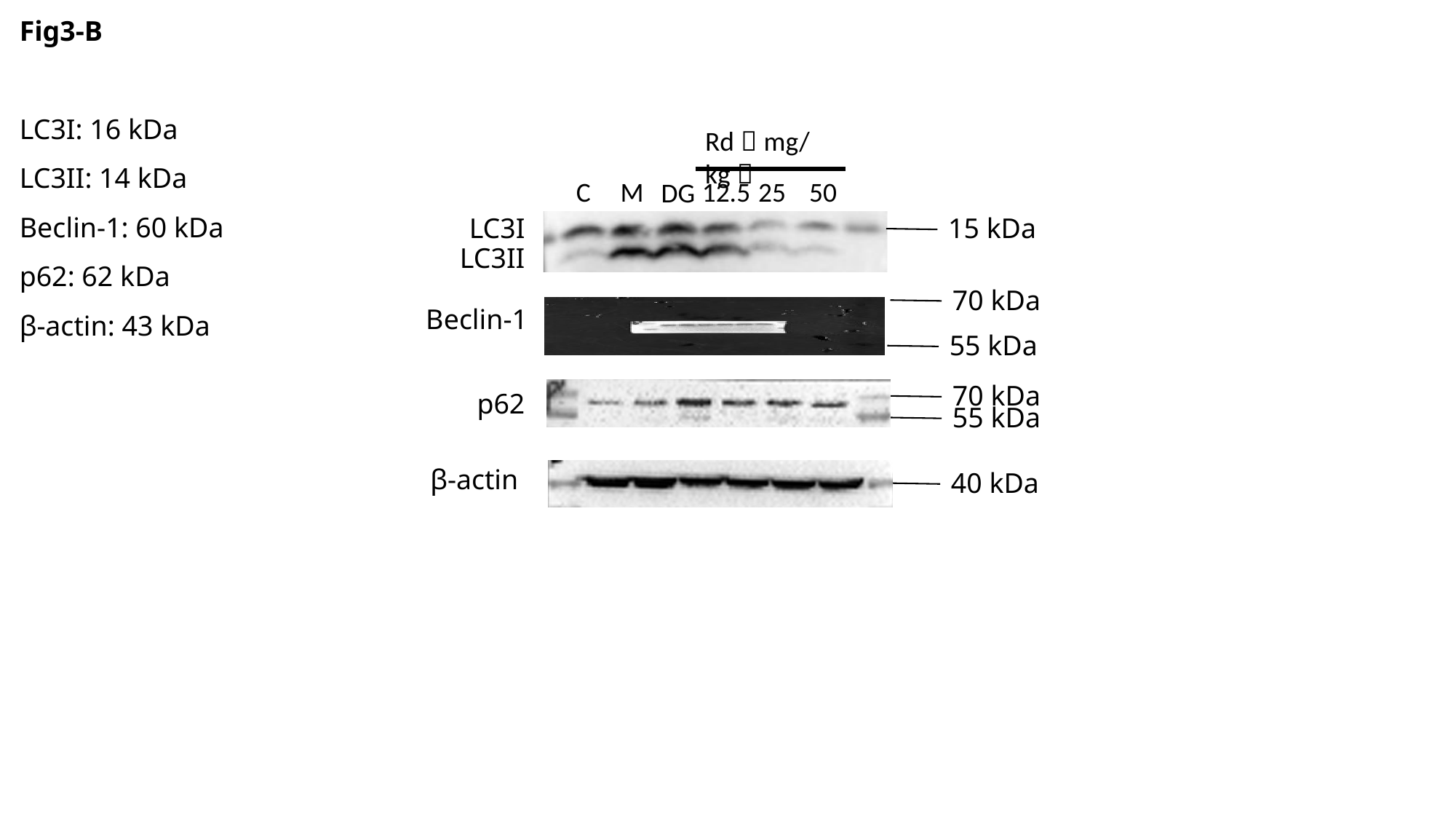

Fig3-B
LC3I: 16 kDa
LC3II: 14 kDa
Beclin-1: 60 kDa
p62: 62 kDa
β-actin: 43 kDa
Rd（mg/kg）
12.5
25
50
C
M
DG
LC3I
15 kDa
LC3II
70 kDa
Beclin-1
55 kDa
70 kDa
p62
55 kDa
β-actin
40 kDa

## Slide 3
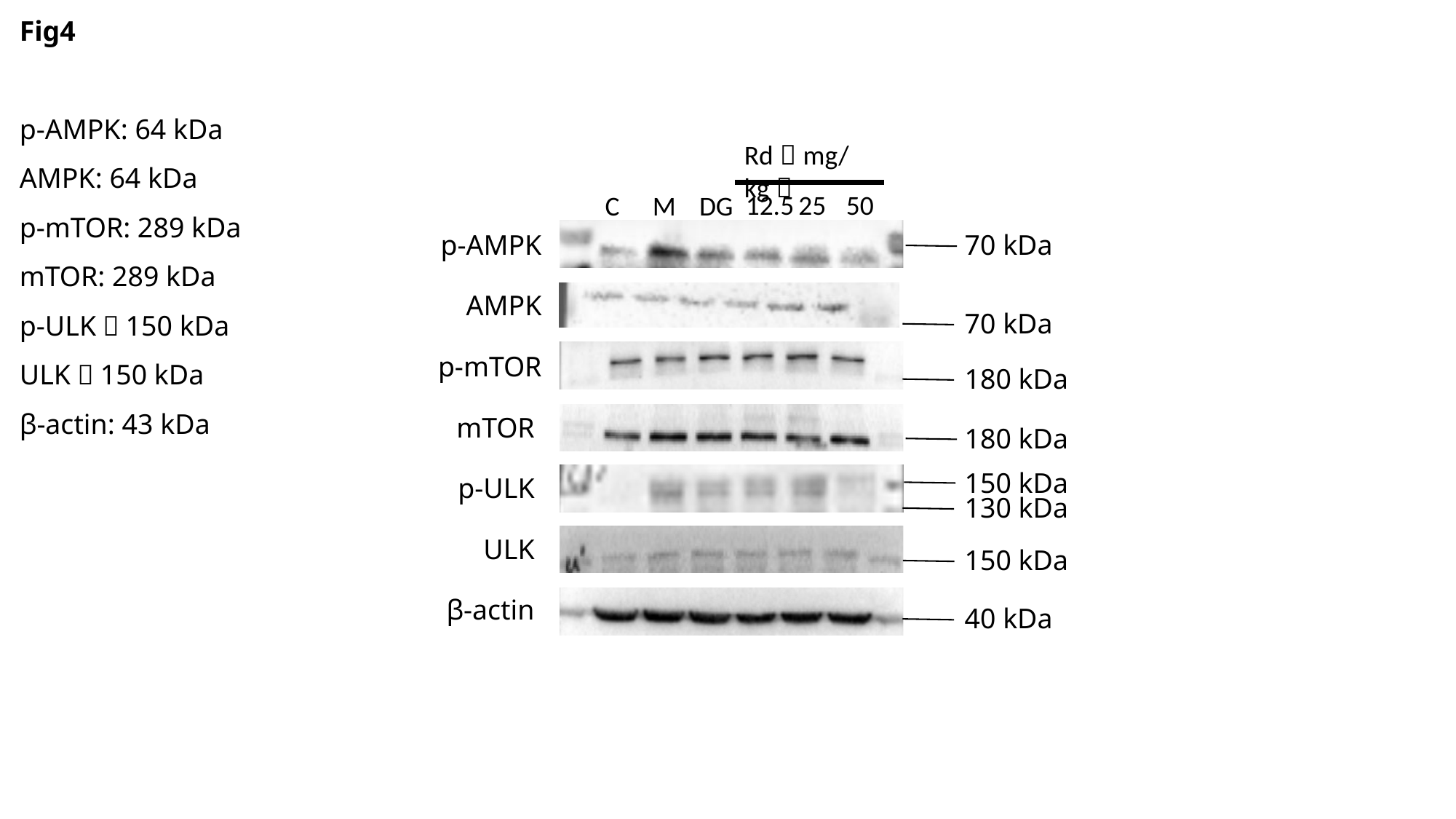

Fig4
p-AMPK: 64 kDa
AMPK: 64 kDa
p-mTOR: 289 kDa
mTOR: 289 kDa
p-ULK：150 kDa
ULK：150 kDa
β-actin: 43 kDa
Rd（mg/kg）
12.5
25
50
C
M
DG
p-AMPK
70 kDa
AMPK
70 kDa
p-mTOR
180 kDa
mTOR
180 kDa
150 kDa
p-ULK
130 kDa
ULK
150 kDa
β-actin
40 kDa

## Slide 4
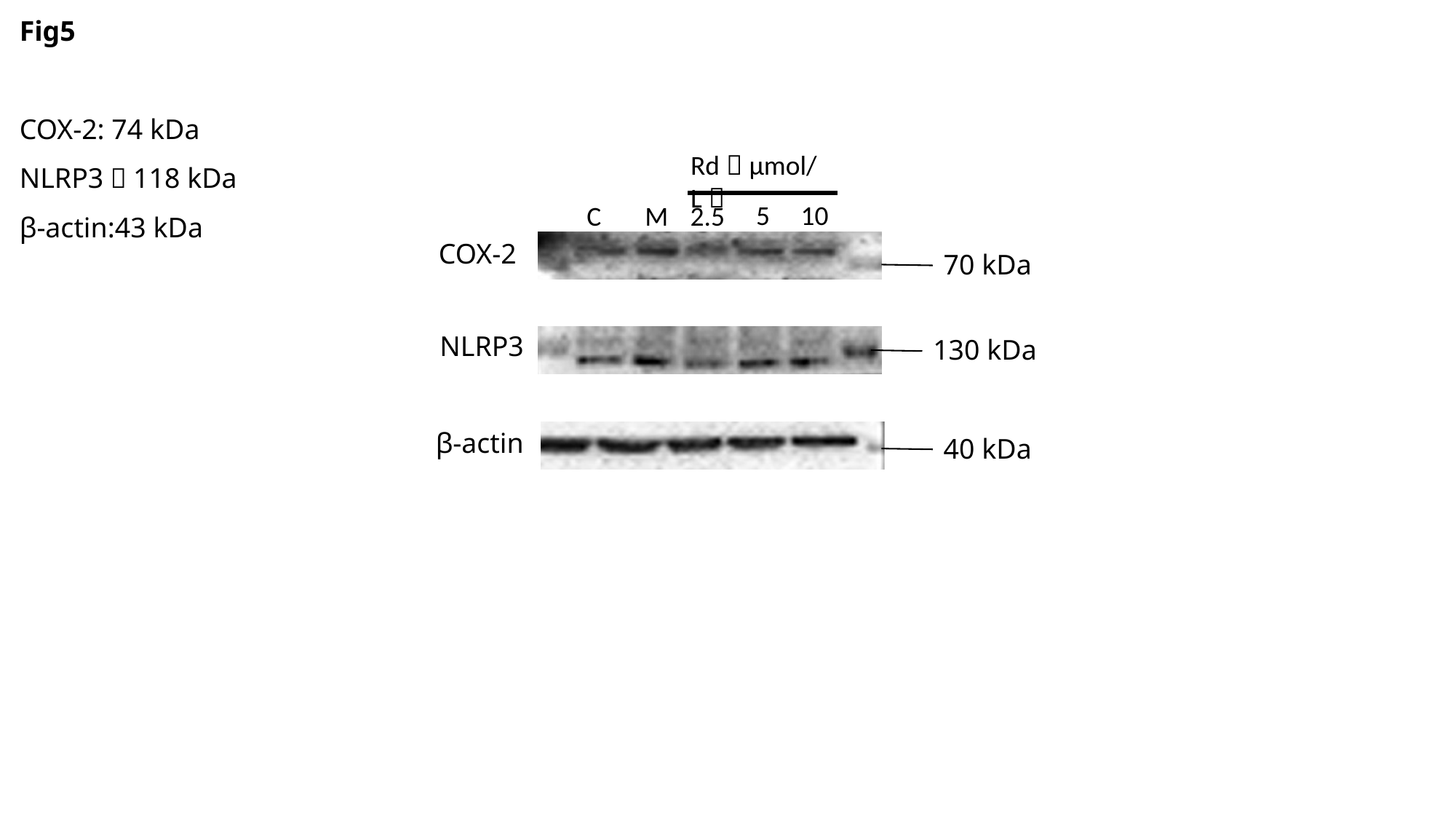

Fig5
COX-2: 74 kDa
NLRP3：118 kDa
β-actin:43 kDa
Rd（μmol/L）
5
10
C
M
2.5
COX-2
70 kDa
NLRP3
130 kDa
β-actin
40 kDa

## Slide 5
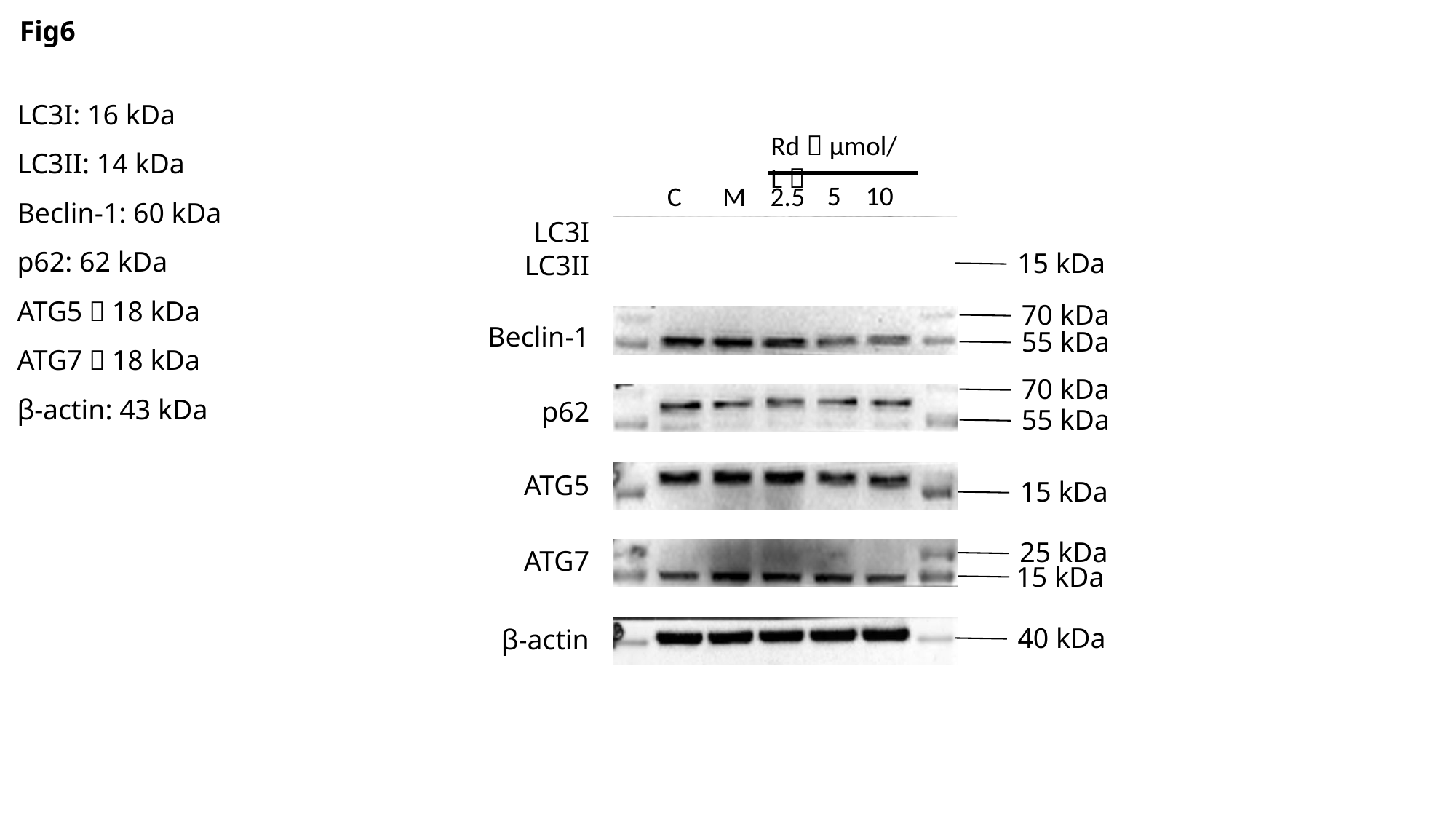

Fig6
LC3I: 16 kDa
LC3II: 14 kDa
Beclin-1: 60 kDa
p62: 62 kDa
ATG5：18 kDa
ATG7：18 kDa
β-actin: 43 kDa
Rd（μmol/L）
5
10
C
M
2.5
LC3I
15 kDa
LC3II
70 kDa
Beclin-1
55 kDa
70 kDa
p62
55 kDa
ATG5
15 kDa
25 kDa
ATG7
15 kDa
40 kDa
β-actin

## Slide 6
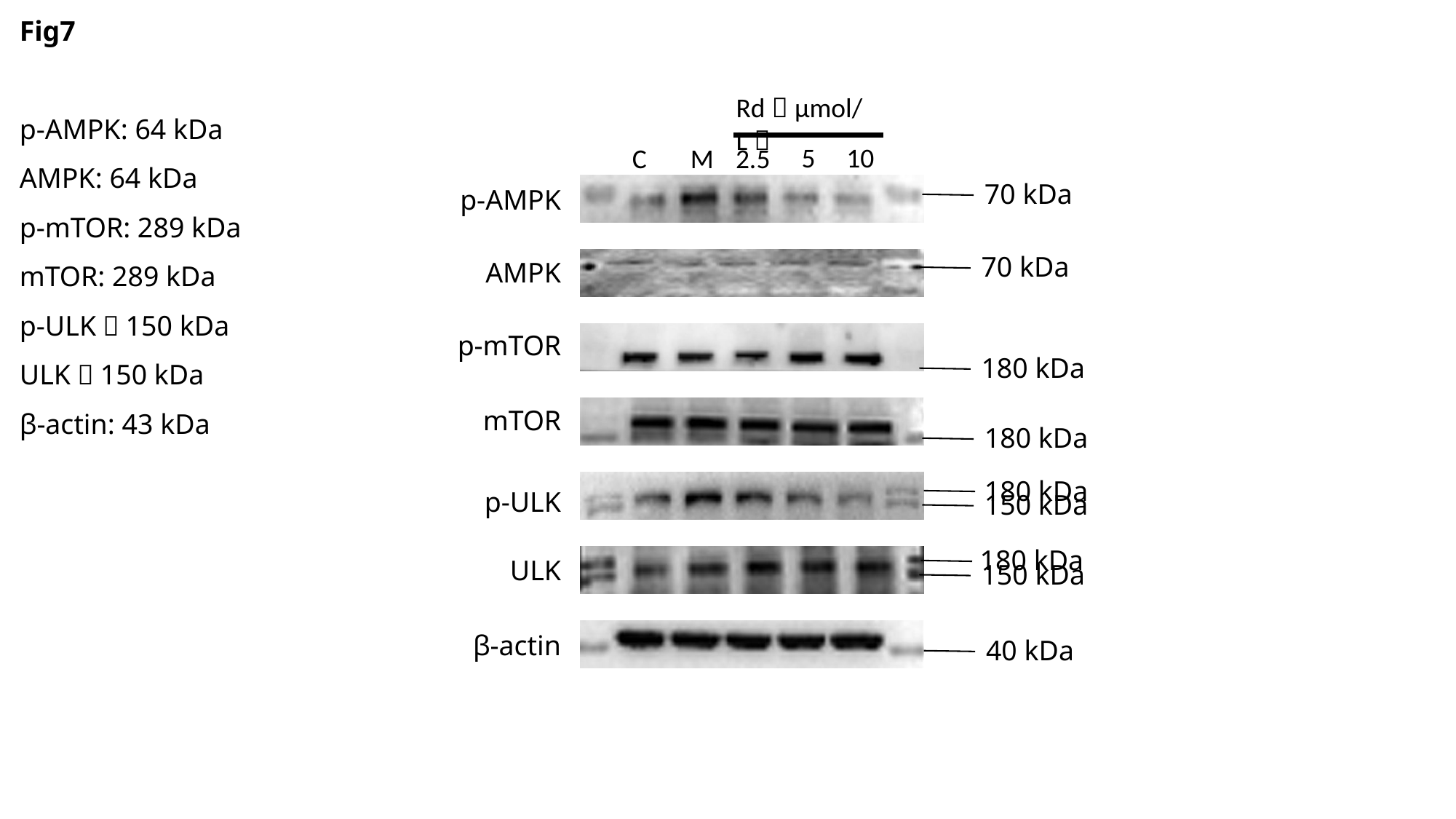

Fig7
p-AMPK: 64 kDa
AMPK: 64 kDa
p-mTOR: 289 kDa
mTOR: 289 kDa
p-ULK：150 kDa
ULK：150 kDa
β-actin: 43 kDa
Rd（μmol/L）
5
10
C
M
2.5
70 kDa
p-AMPK
70 kDa
AMPK
p-mTOR
180 kDa
mTOR
180 kDa
180 kDa
p-ULK
150 kDa
180 kDa
ULK
150 kDa
β-actin
40 kDa

## Slide 7
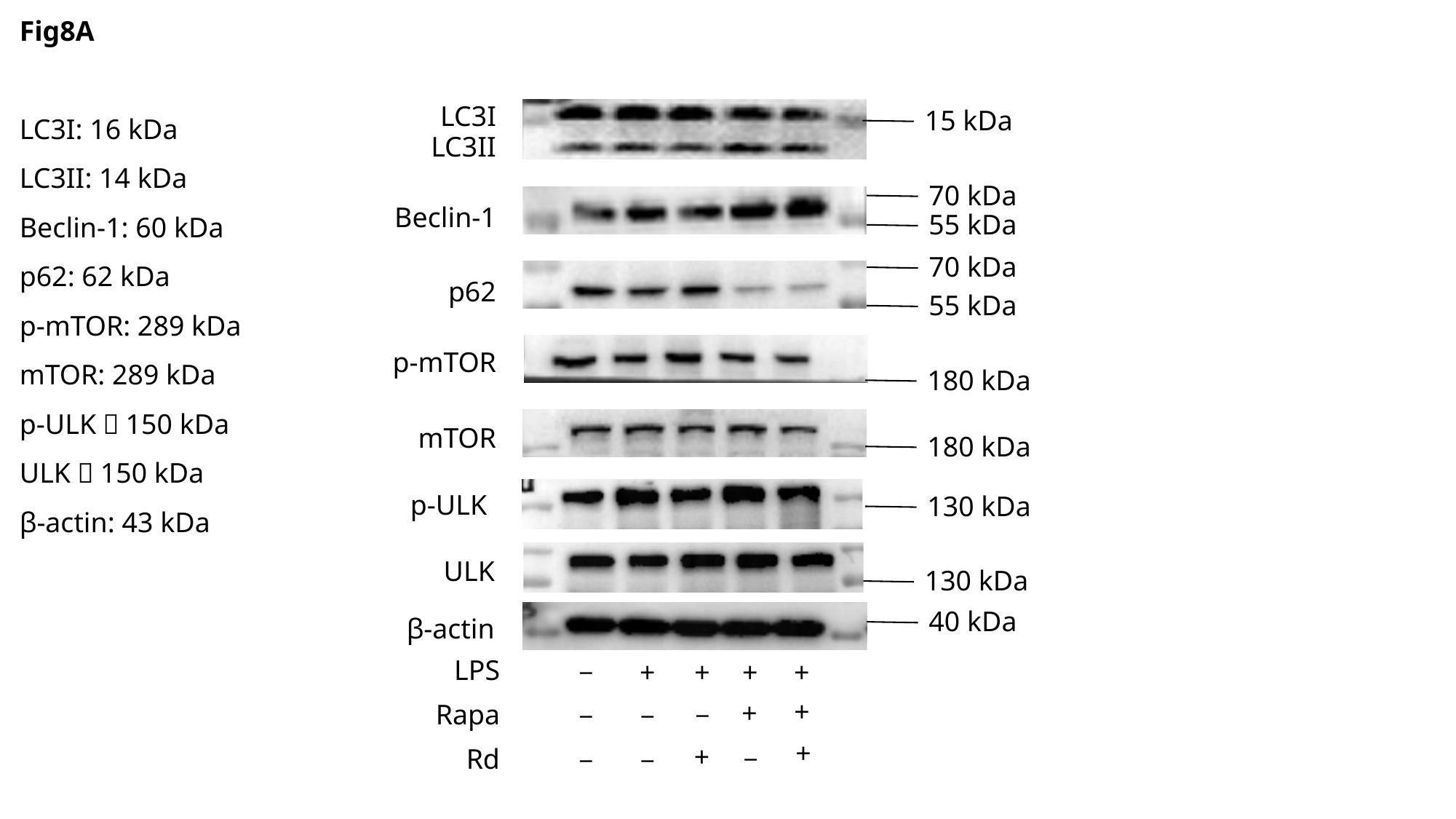

Fig8A
LC3I: 16 kDa
LC3II: 14 kDa
Beclin-1: 60 kDa
p62: 62 kDa
p-mTOR: 289 kDa
mTOR: 289 kDa
p-ULK：150 kDa
ULK：150 kDa
β-actin: 43 kDa
LC3I
15 kDa
LC3II
70 kDa
Beclin-1
55 kDa
70 kDa
p62
55 kDa
p-mTOR
180 kDa
mTOR
180 kDa
p-ULK
130 kDa
ULK
130 kDa
40 kDa
β-actin
_
LPS
+
+
+
+
_
_
_
+
+
Rapa
_
_
_
+
+
Rd

## Slide 8
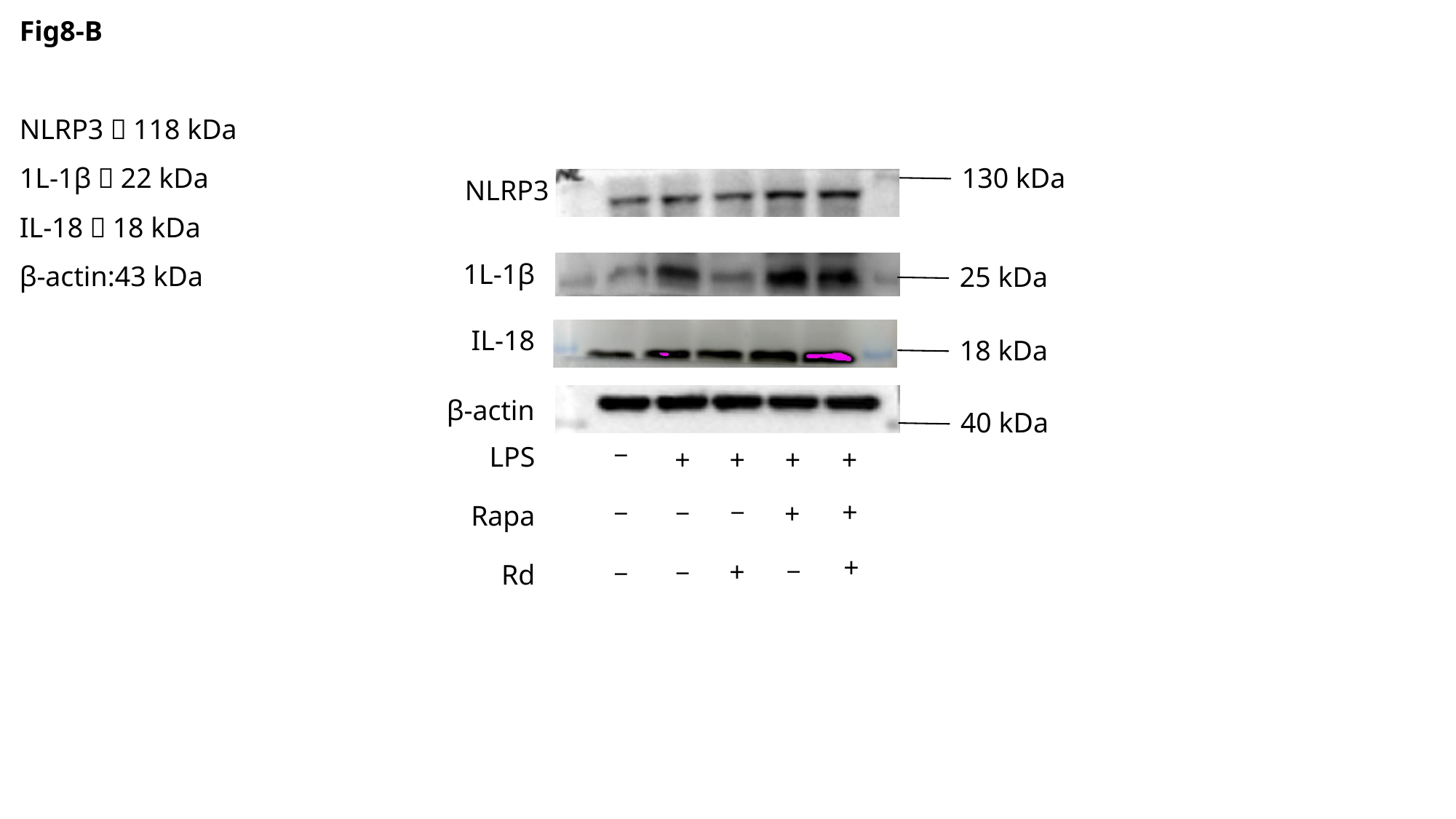

Fig8-B
NLRP3：118 kDa
1L-1β：22 kDa
IL-18：18 kDa
β-actin:43 kDa
130 kDa
NLRP3
1L-1β
25 kDa
IL-18
18 kDa
β-actin
40 kDa
_
LPS
+
+
+
+
_
_
_
+
+
Rapa
_
_
_
+
+
Rd

## Slide 9
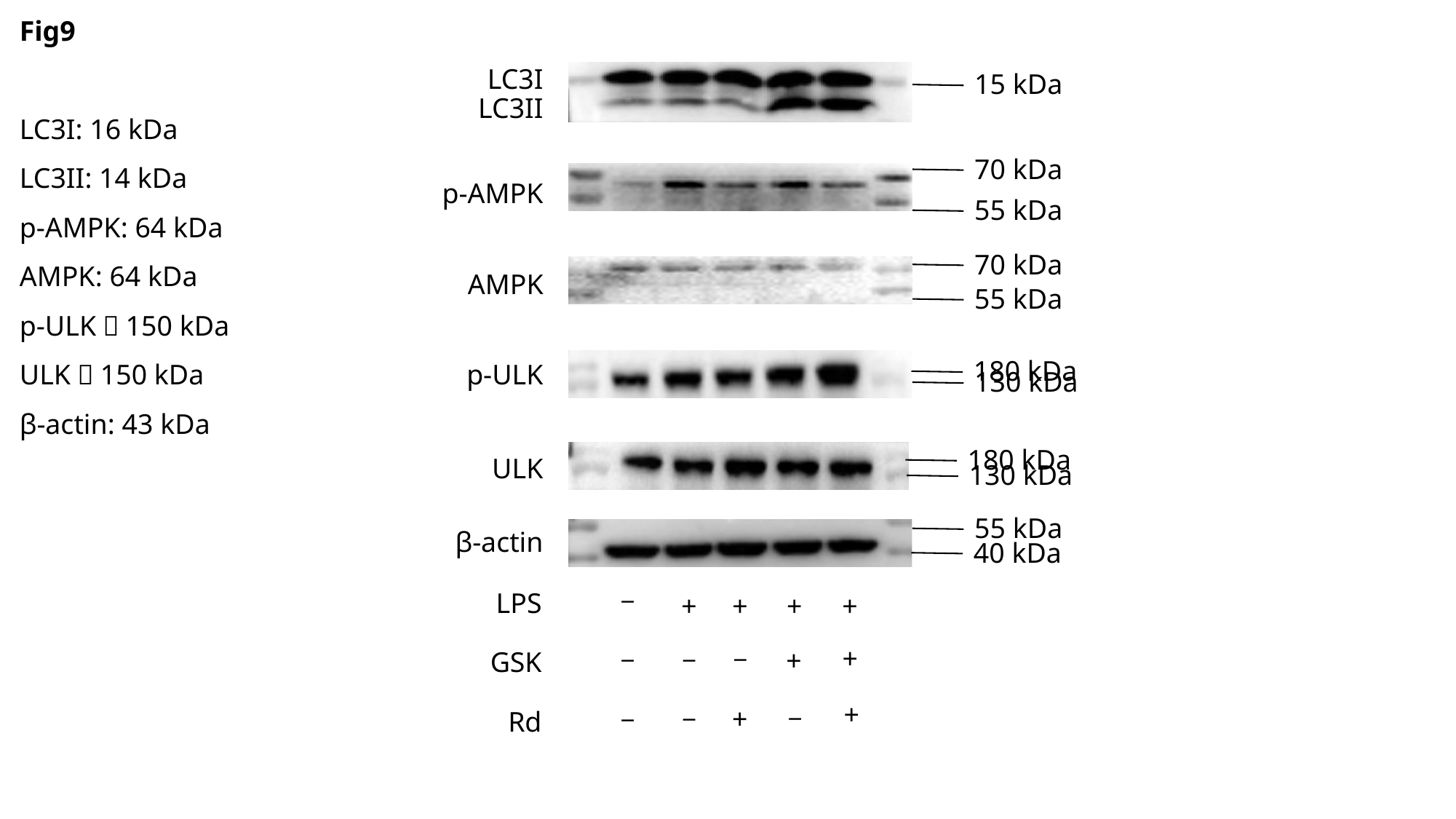

Fig9
LC3I: 16 kDa
LC3II: 14 kDa
p-AMPK: 64 kDa
AMPK: 64 kDa
p-ULK：150 kDa
ULK：150 kDa
β-actin: 43 kDa
LC3I
15 kDa
LC3II
70 kDa
p-AMPK
55 kDa
70 kDa
AMPK
55 kDa
180 kDa
p-ULK
130 kDa
180 kDa
ULK
130 kDa
55 kDa
β-actin
40 kDa
_
LPS
+
+
+
+
_
_
_
+
+
GSK
_
_
_
+
+
Rd
