# Supplementary material for: Ginsenoside Rd protects against acute liver injury by regulating the autophagy NLRP3 inflammasome pathway
Source: Sci Rep. 2025 Jan 28;15:3569. doi: 10.1038/s41598-025-87991-9 (PMC11775168; doi:10.1038/s41598-025-87991-9)
Supplement: Supplementary file 2 — Supplementary Material 2 [file 41598_2025_87991_MOESM2_ESM.docx]

**Table 1. The primer sequences used in qPCR analysis.**

| Primers | Forward (5’→3’) | Reverse (5’→3’) |
| --- | --- | --- |
| IL-6 | CTGCAAGAGACTTCCATCCAG | AGTGGTATAGACAGGTCTGTTGG |
| TNF-α | GCCGATGGGTTGTACCTTGT | TCTTGACGGCAGAGAGGAGG |
| iNOS | GAAGGGGACGAACTCAGTGG | GTGGCTCCCATGTTGCATTG |
| COX-2 | GCCTGGTCTGATGATGTATGC | CCTATGAGTATGAGTCTGCTGGTT |
| β-actin | TGTCCACCTTCCAGCAGATGT | AGCTCAGTAACAGTCCGCCTAG |


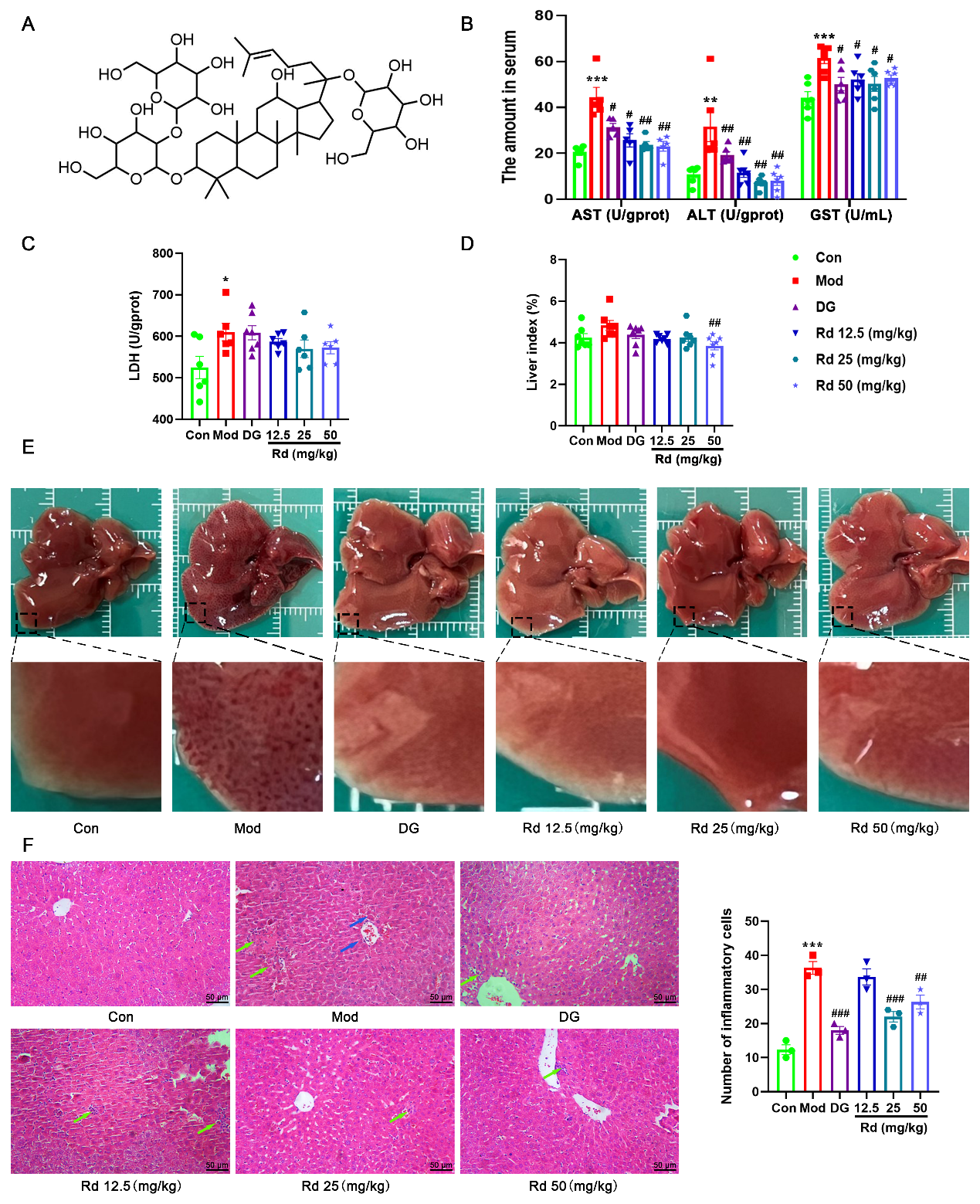


**Figure 1: Rd ameliorated TAA-induced acute liver injury in mice.** A) chemical structure of Rd. B, C) Effect of Rd on AST, ALT, LDH and GST levels in serum of liver-injured mice (n = 6). D) Effect of Rd on liver index in mice with acute liver injury (n = 6). E) Effect of Rd on the appearance of liver tissue in mice (n = 6). F) Effect of Rd on pathological changes and number of inflammatory cells in mouse liver histological sections (200×, n = 3). Red arrows indicated inflammatory cell infiltration. The blue arrow indicates nuclear condensation. Values are shown as the mean ± SEM; **p* < 0.05, ***p* < 0.01, ****p* < 0.001 vs. control group, # *p* <0.05, ##*p* < 0.01, ###*p* <0.001 vs. model group, analyzed by one-way ANOVA with Dunnett’s test.

**
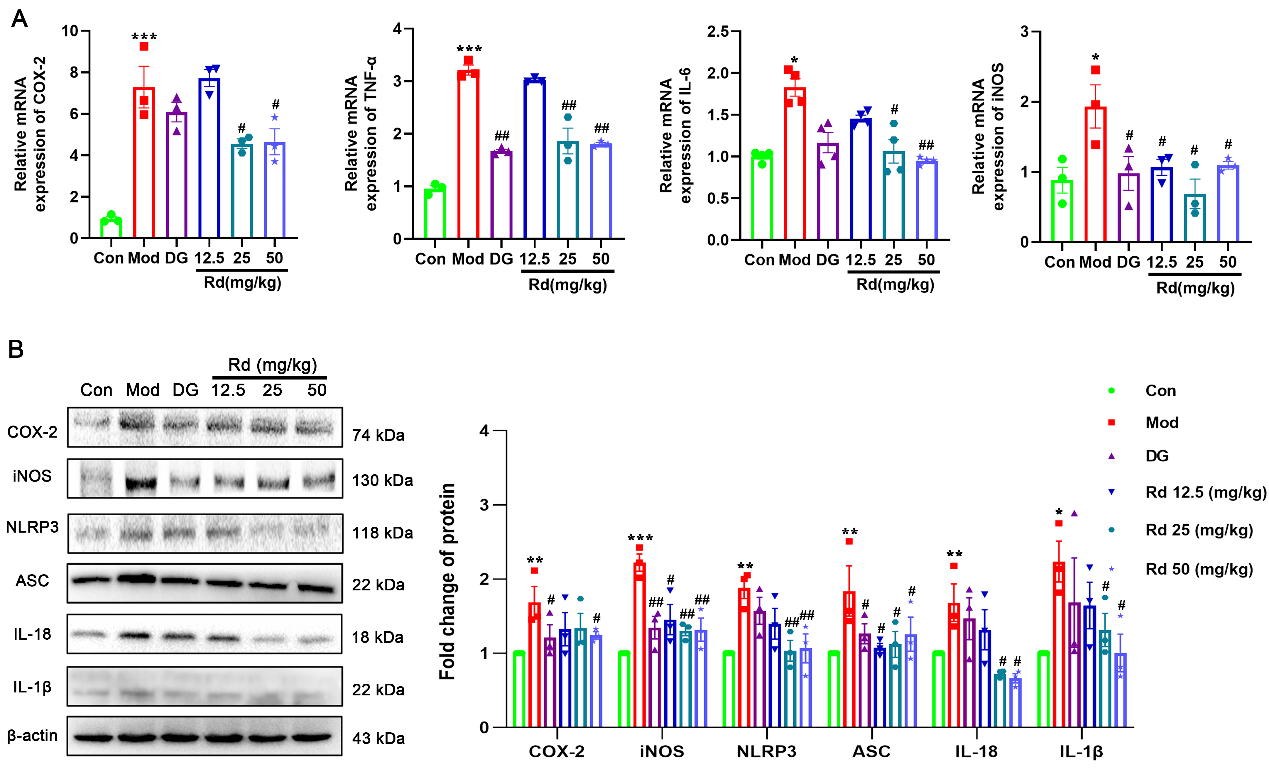
**

**Figure 2: Rd alleviated hepatic tissue inflammation in mice with acute liver injury.** A) Effect of Rd on liver tissue COX-2 TNF-α, IL-6, iNOS, mRNA expression effect (n = 3). B) Effect of Rd on liver tissue COX-2, iNOS, NLRP3, ASC, IL-18, IL-1β in liver-injured mice (n = 3). Values are shown as the mean ± SEM; **p* < 0.05, ***p* < 0.01, ****p* < 0.001 vs. control group, #*p* < 0.05, ##*p* < 0.01 vs. model group, analyzed by one-way ANOVA with Dunnett’s test.


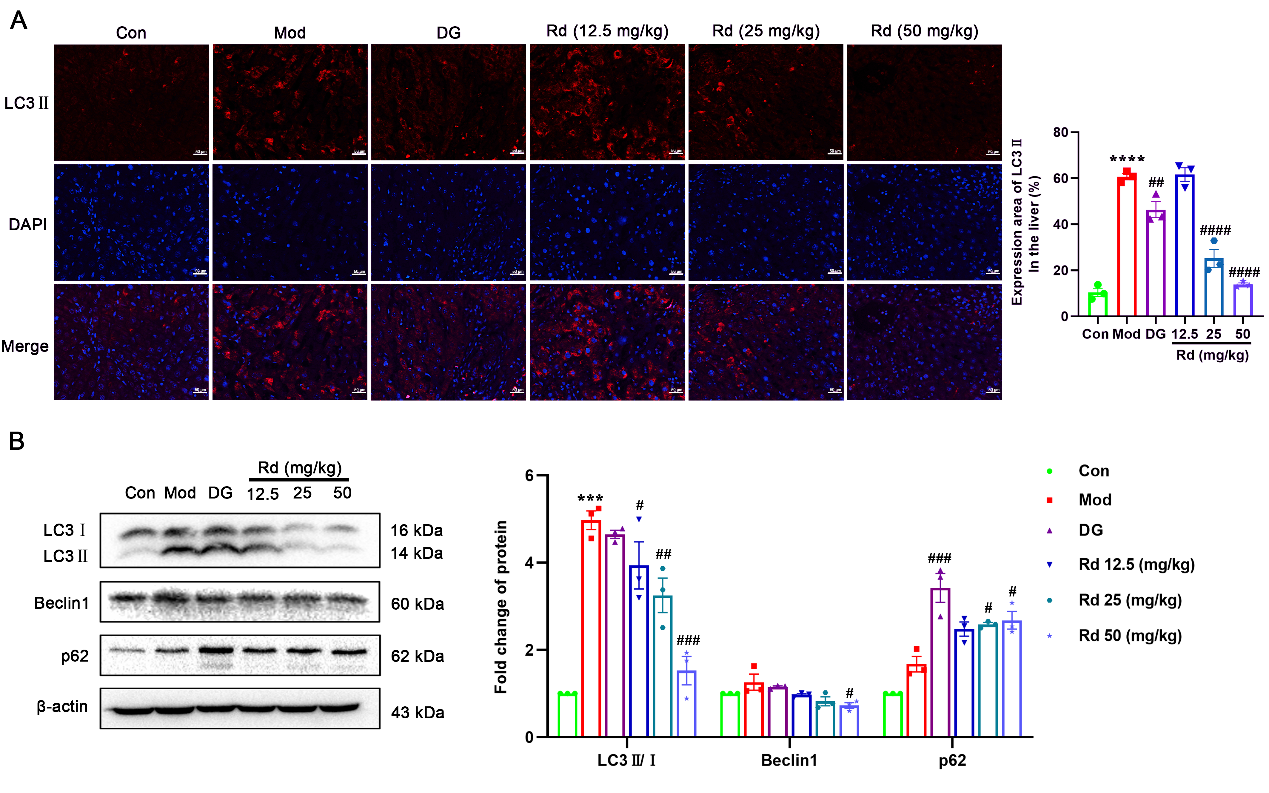


**Figure 3: Rd regulates hepatic tissue autophagy levels in mice with acute liver injury.** A) Effect of Rd on LC3II protein expression in liver tissues of liver-injured mice (400×, n = 3). B) Effects of Rd on liver tissues of mice with acute liver injury LC3, Beclin1, p62 protein expression levels in mice with acute liver injury (n = 3).Values are shown as the mean ± SEM, ****p* < 0.001, *****p* < 0.0001 vs. control group, #*p* < 0.05, ##*p* < 0.01, ###*p* < 0.001, ####*p* < 0.0001vs. model group, analyzed by one-way ANOVA with Dunnett’s test.


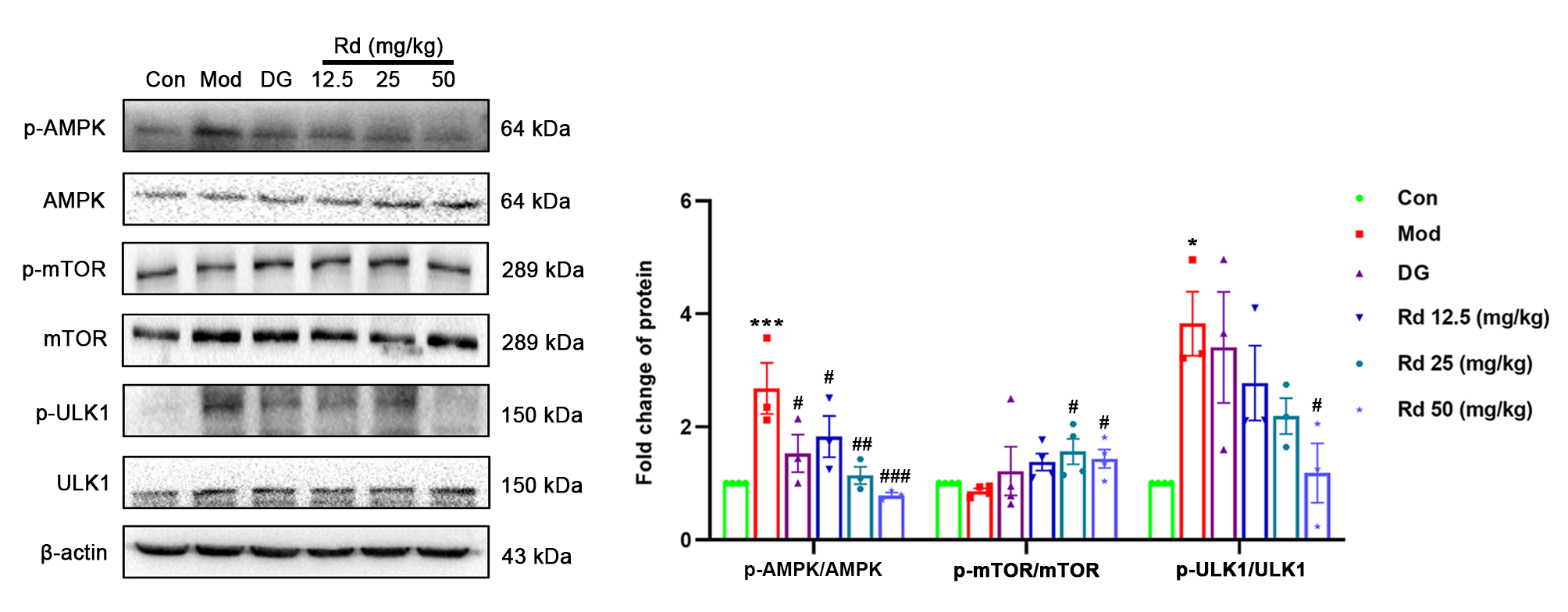


**Figure 4: Rd regulates hepatic tissue AMPK pathway in mice with acute liver injury.** Effects of Rd on the liver tissues of mice with acute liver injury on AMPK, mTOR, ULK1 phosphorylation levels in mice with acute liver injury (n = 3). Values are shown as the mean ± SEM; **p* < 0.05, ****p* < 0.001, vs. control group, #*p* < 0.05, ##*p* < 0.01, ###*p* < 0.001, vs. model group, analyzed by one-way ANOVA with Dunnett’s test.


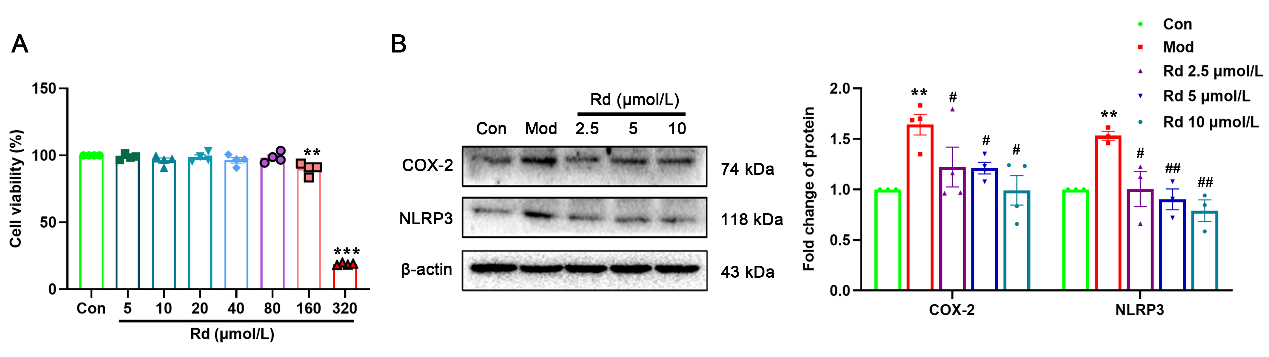


**Figure 5: Rd regulates LPS-induced inflammation in HSC-T6 cells.** A) Effect of incubation with different concentrations of Rd for 24 h on the viability of HSC-T6 cells (n = 4). B) Effect of Rd on LPS-induced inflammation-associated protein expression in HSC-T6 cells (n = 3). Values are shown as the mean ± SEM, ***p* < 0.01, ****p* < 0.001 vs. control group, #*p* < 0.05, ##*p* < 0.01 vs. model group, analyzed by one-way ANOVA with Dunnett’s test.


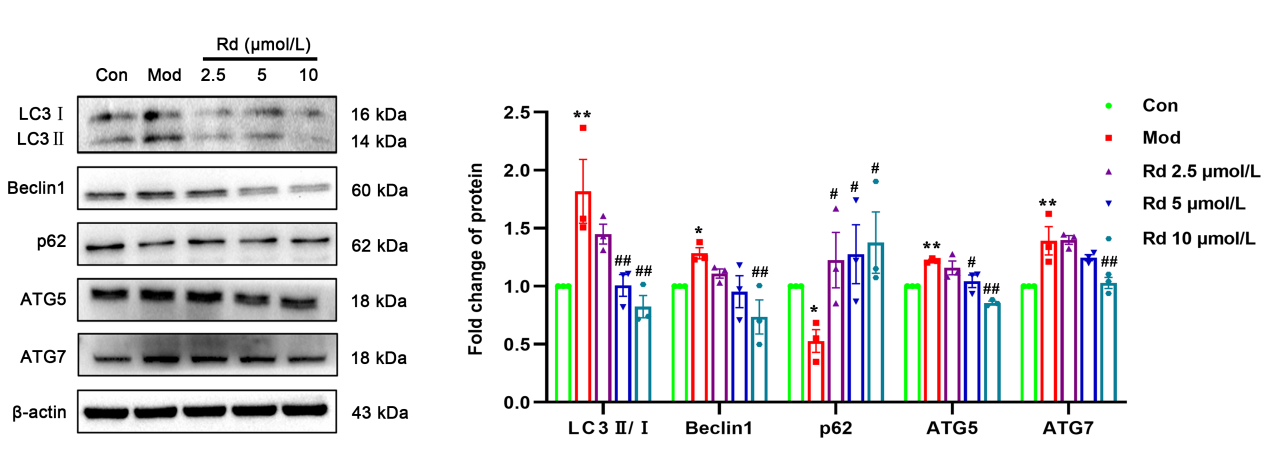


**Figure 6: Rd regulates LPS-induced autophagy in HSC-T6 cells.** Effect of Rd on LPS-induced autophagy-related protein expression in HSC-T6 cells (n = 3). Values are shown as the mean ± SEM; **p* < 0.05, ***p* < 0.01, vs. control group, #*p* < 0.05, ##*p* < 0.01 vs. model group, analyzed by one-way ANOVA with Dunnett’s test.


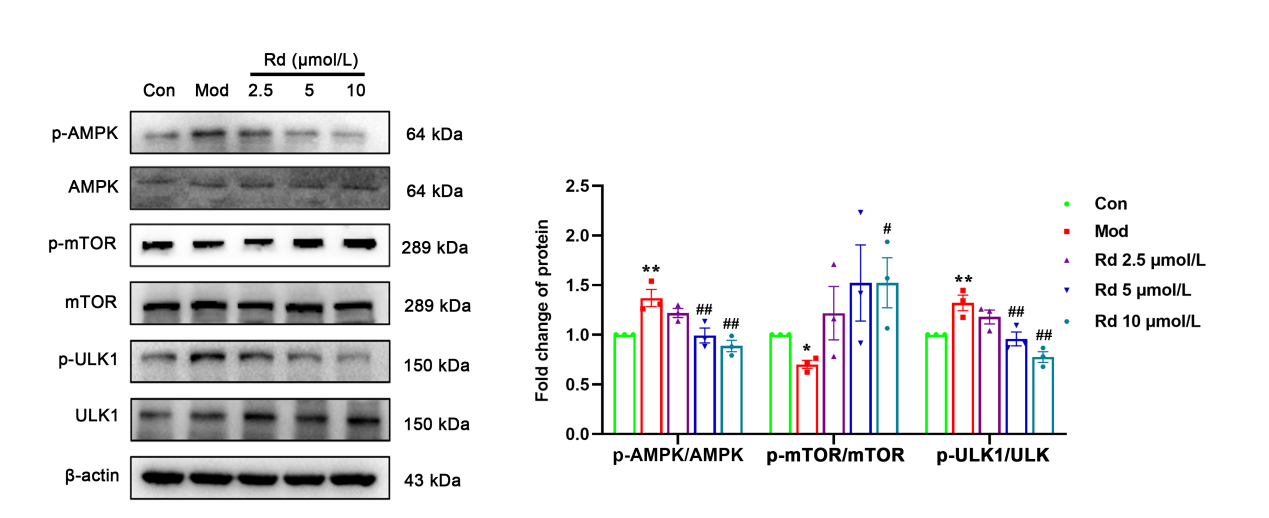


**Figure 7: Rd regulates AMPK pathway in LPS-induced HSC-T6 cells.** Effect of Rd on AMPK/mTOR/ULK1 pathway in HSC-T6 cells Effect of Rd on AMPK/mTOR/ULK1 pathway in HSC-T6 cells (n = 3). Values are shown as the mean ± SEM; **p* < 0.05, ***p* < 0.01, vs. control group, #*p* < 0.05, ##*p* < 0.01 vs. model group, analyzed by one-way ANOVA with Dunnett’s test.


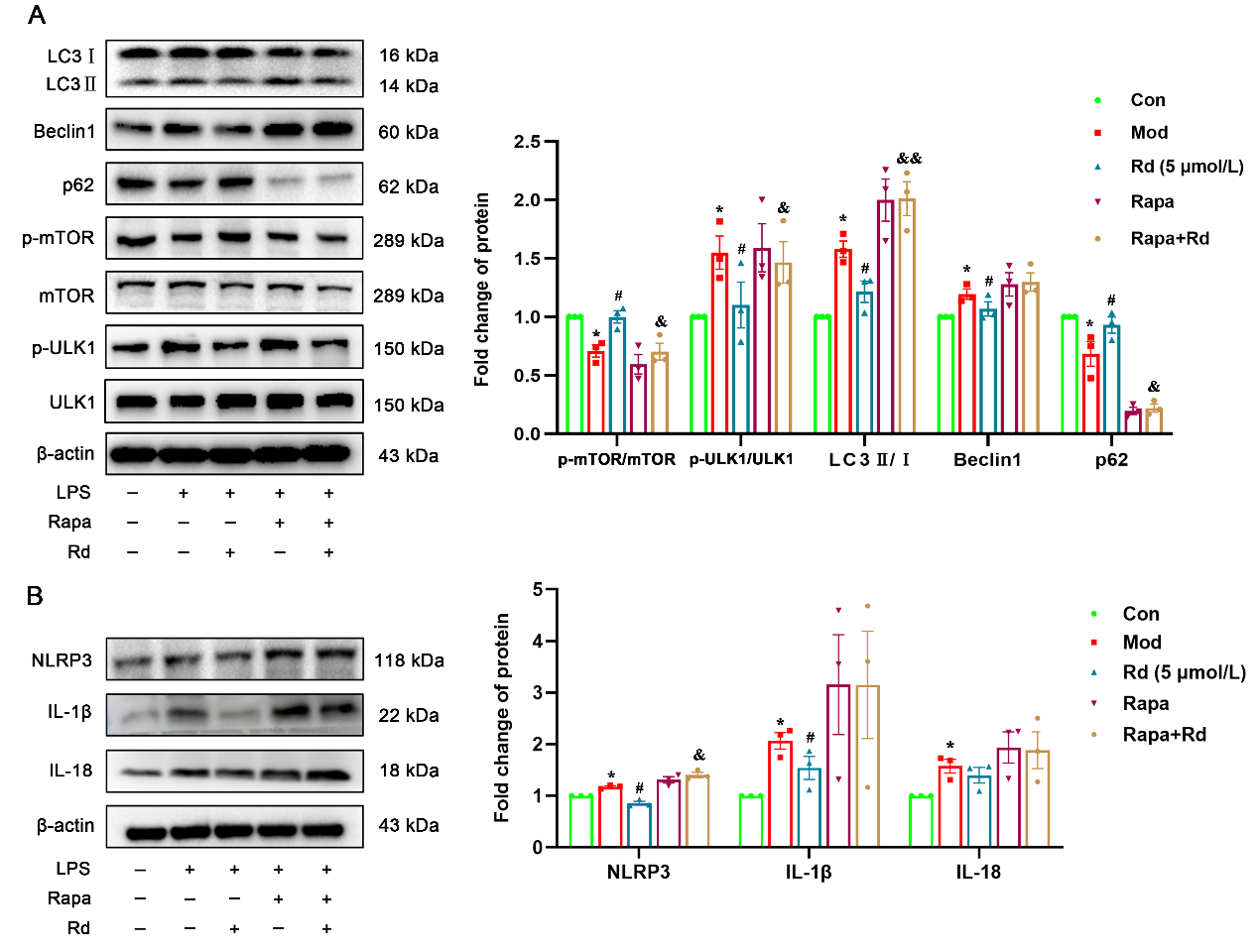


**Figure 8: Intervention of Rapamycin on autophagy regulation by Rd.** A) Effect of Rapamycin intervention on the regulation of autophagy in HSC-T6 cells by Rd (n = 3). B) Effect of Rd on Rapamycin-intervened HSC-T6 cell inflammation-associated protein expression (n = 3). Values are shown as the mean ± SEM; **p* < 0.05 vs. control group, #*p* < 0.05 vs. model group, &*p* < 0.05, &&*p* < 0.01 vs. treatment group, analyzed by one-way ANOVA with Dunnett’s test.


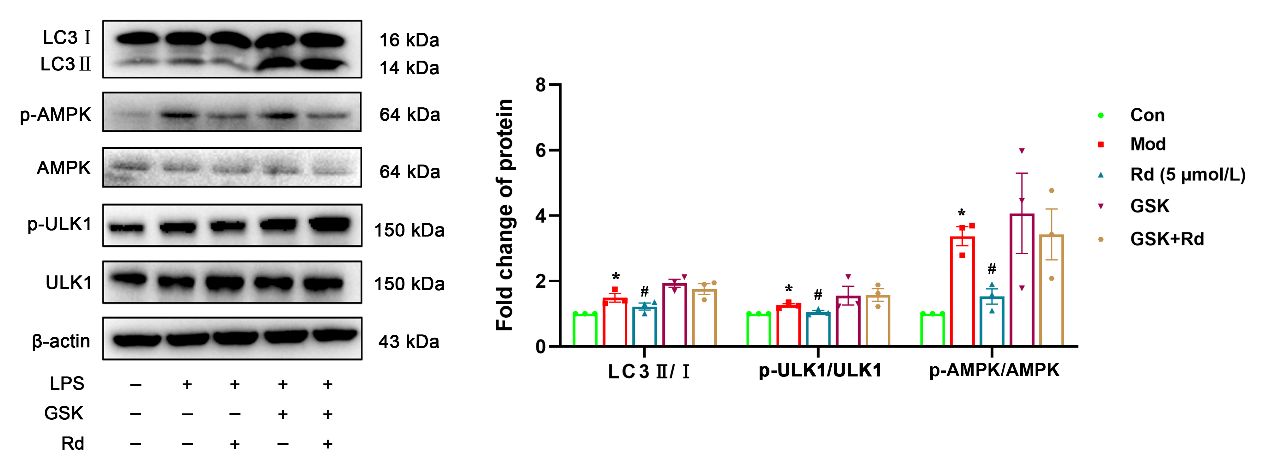


**Figure 9: Intervention of GSK621 on autophagy regulation by Rd.** Effect of GSK621 intervention on the effect of Rd on regulating autophagy in HSC-T6 cells (n = 3). Values are shown as the mean ± SEM; **p* < 0.05 vs. control group, #*p* < 0.05 vs. model group, analyzed by one-way ANOVA with Dunnett’s test.
