# Supplementary material for: Ginsenoside Rd protects against acute liver injury by regulating the autophagy NLRP3 inflammasome pathway
Source: Sci Rep. 2025 Jan 28;15:3569. doi: 10.1038/s41598-025-87991-9 (PMC11775168; doi:10.1038/s41598-025-87991-9)

Fig2-B

COX-2: 74 kDa

iNOS: 130 kDa

NLRP3： 118 kDa

ASC： 22 kDa

IL-18： 18 kDa

1L-1β： 22 kDa

β-actin: 43 kDa

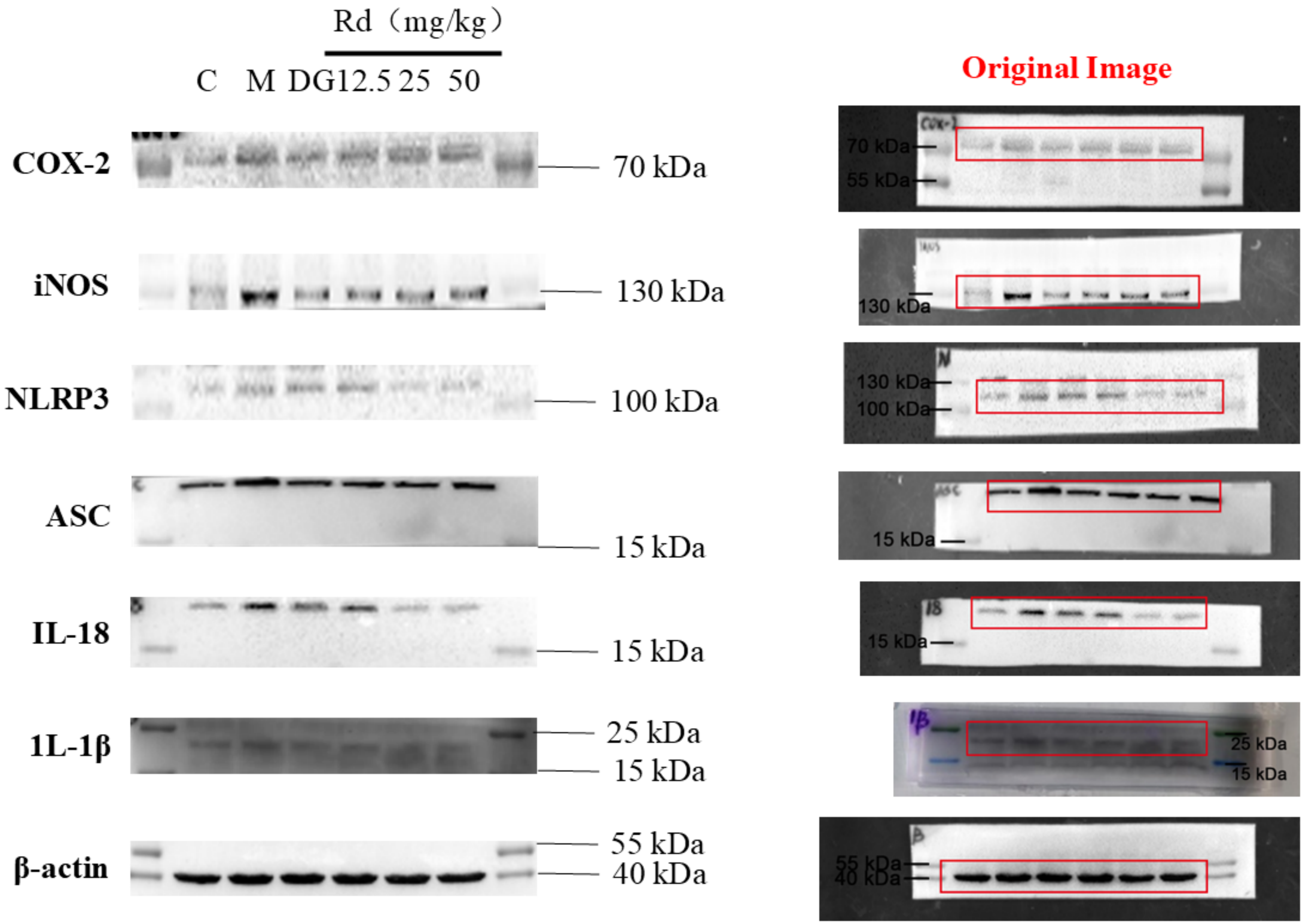

Fig3-B

LC3I: 16 kDa

LC3II: 14 kDa

Beclin-1: 60 kDa

p62: 62 kDa

β-actin: 43 kDa

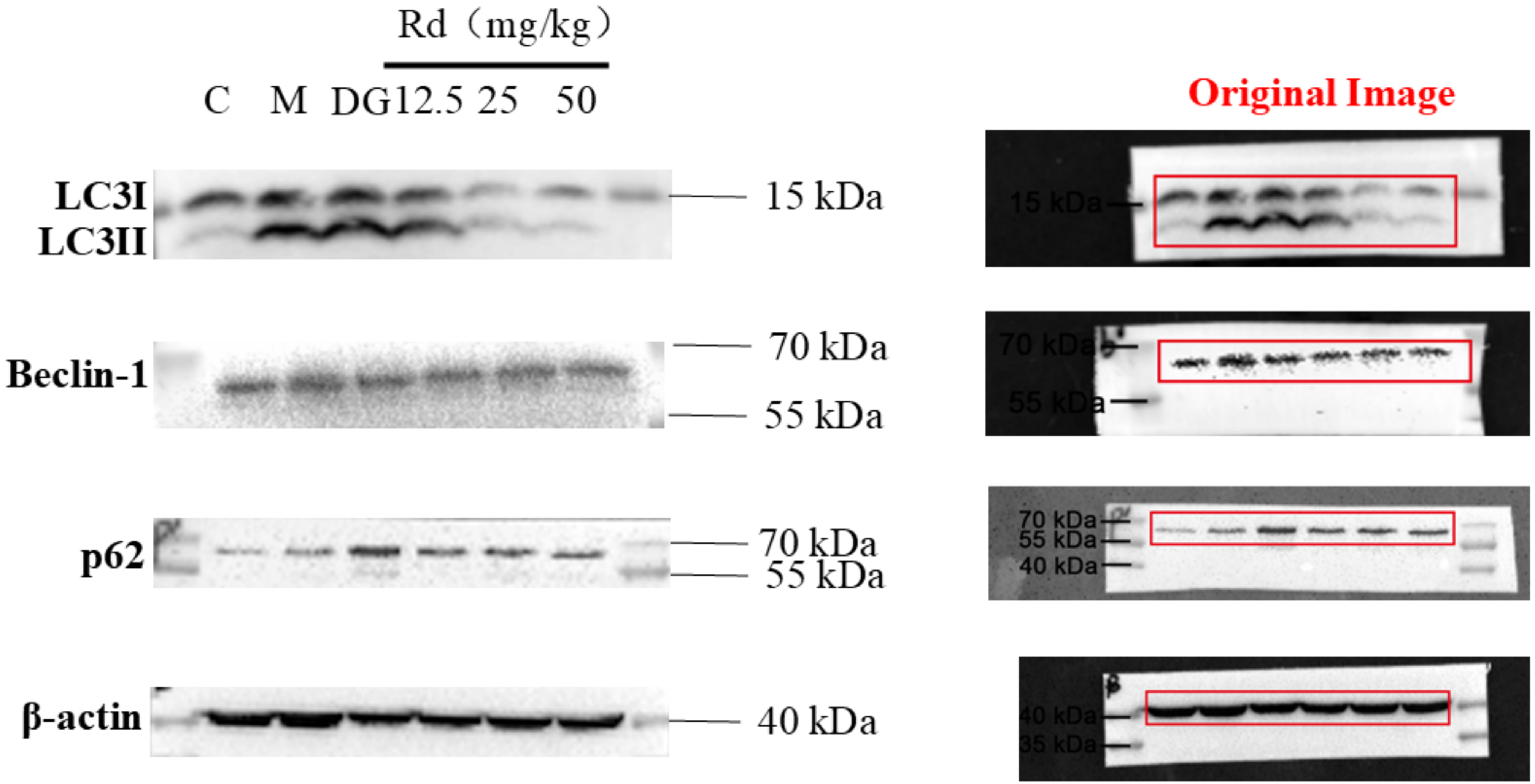

Fig4

p-AMPK: 64 kDa

AMPK: 64 kDa

p-mTOR: 289 kDa

mTOR: 289 kDa

p-ULK： 150 kDa

ULK： 150 kDa

β-actin: 43 kDa

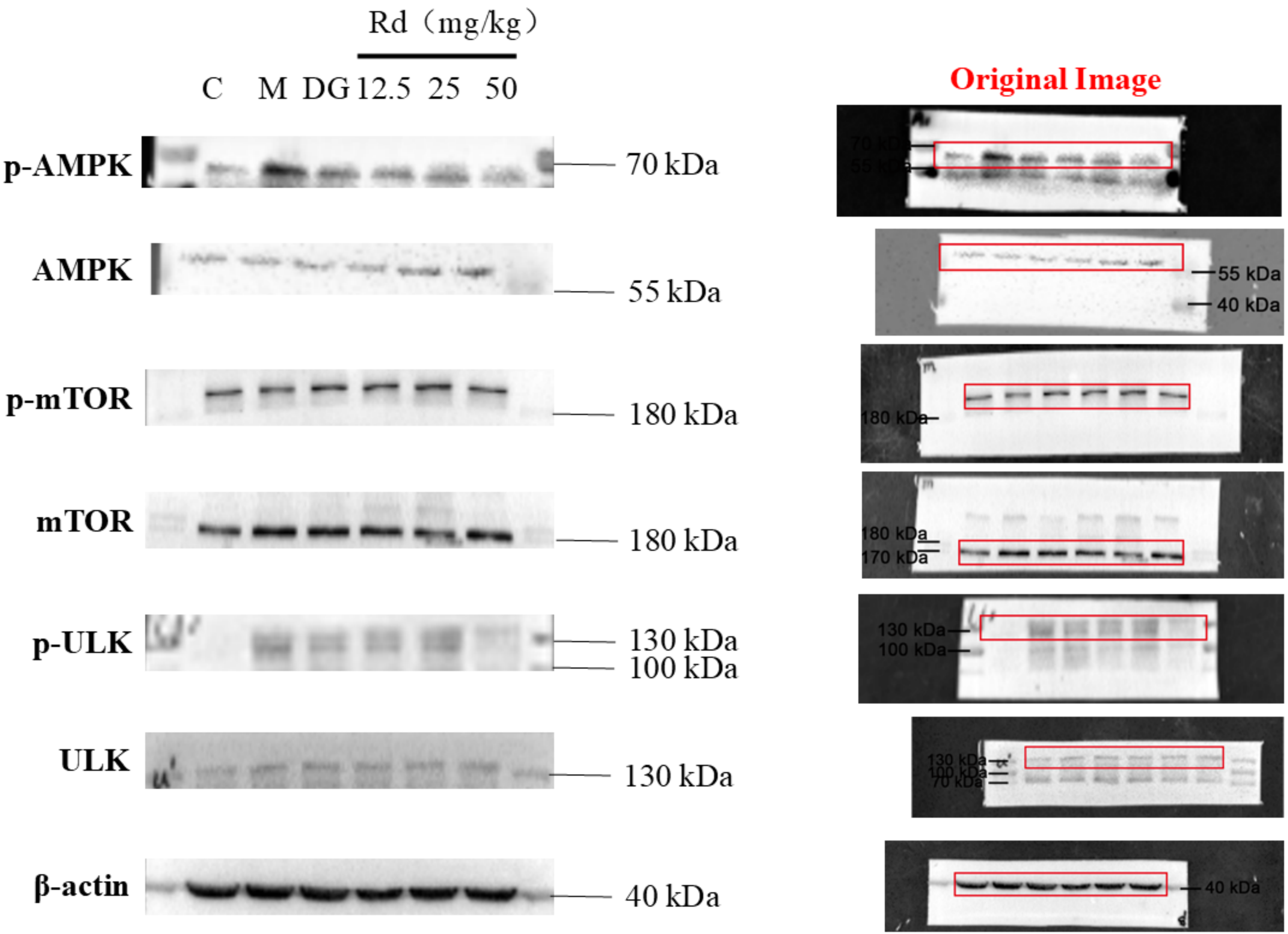

Fig5

COX-2: 74 kDa

NLRP3： 118 kDa

β-actin:43 kDa

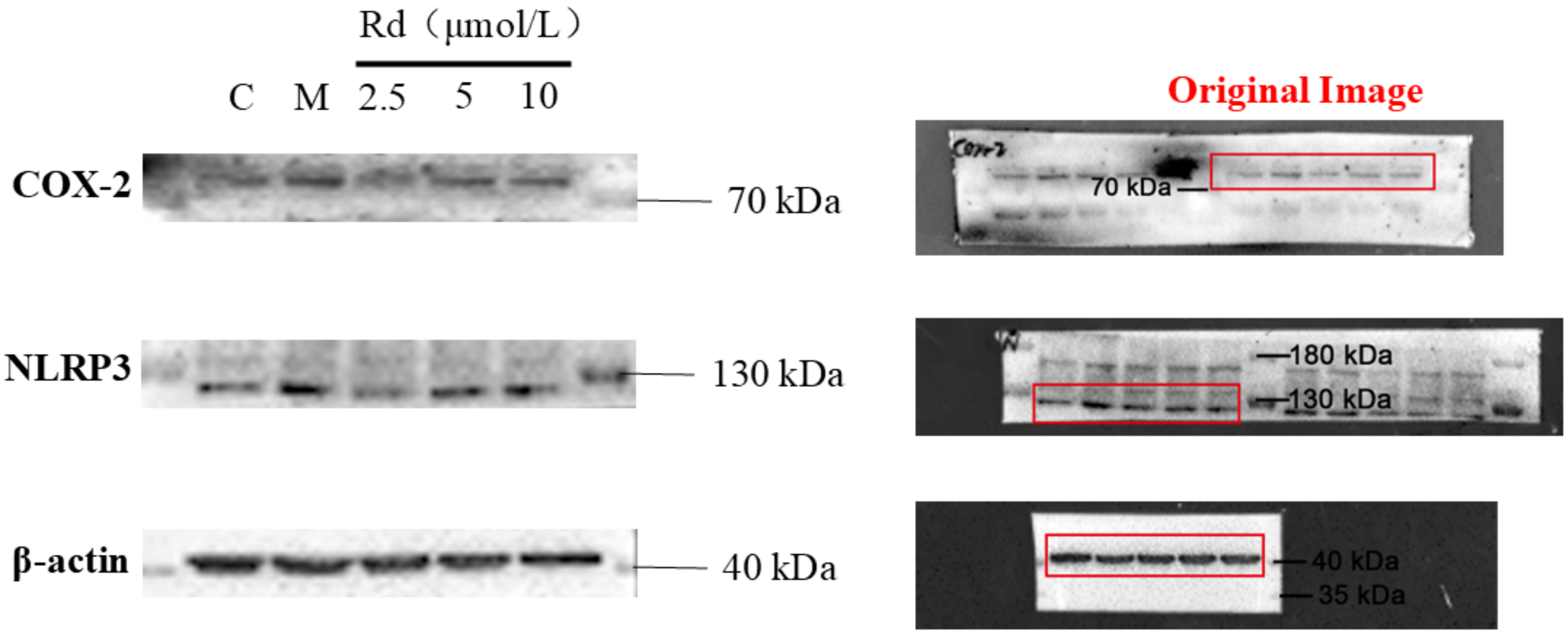

Fig6

LC3I: 16 kDa

LC3II: 14 kDa

Beclin-1: 60 kDa

p62: 62 kDa

ATG5： 18 kDa

ATG7： 18 kDa

β-actin: 43 kDa

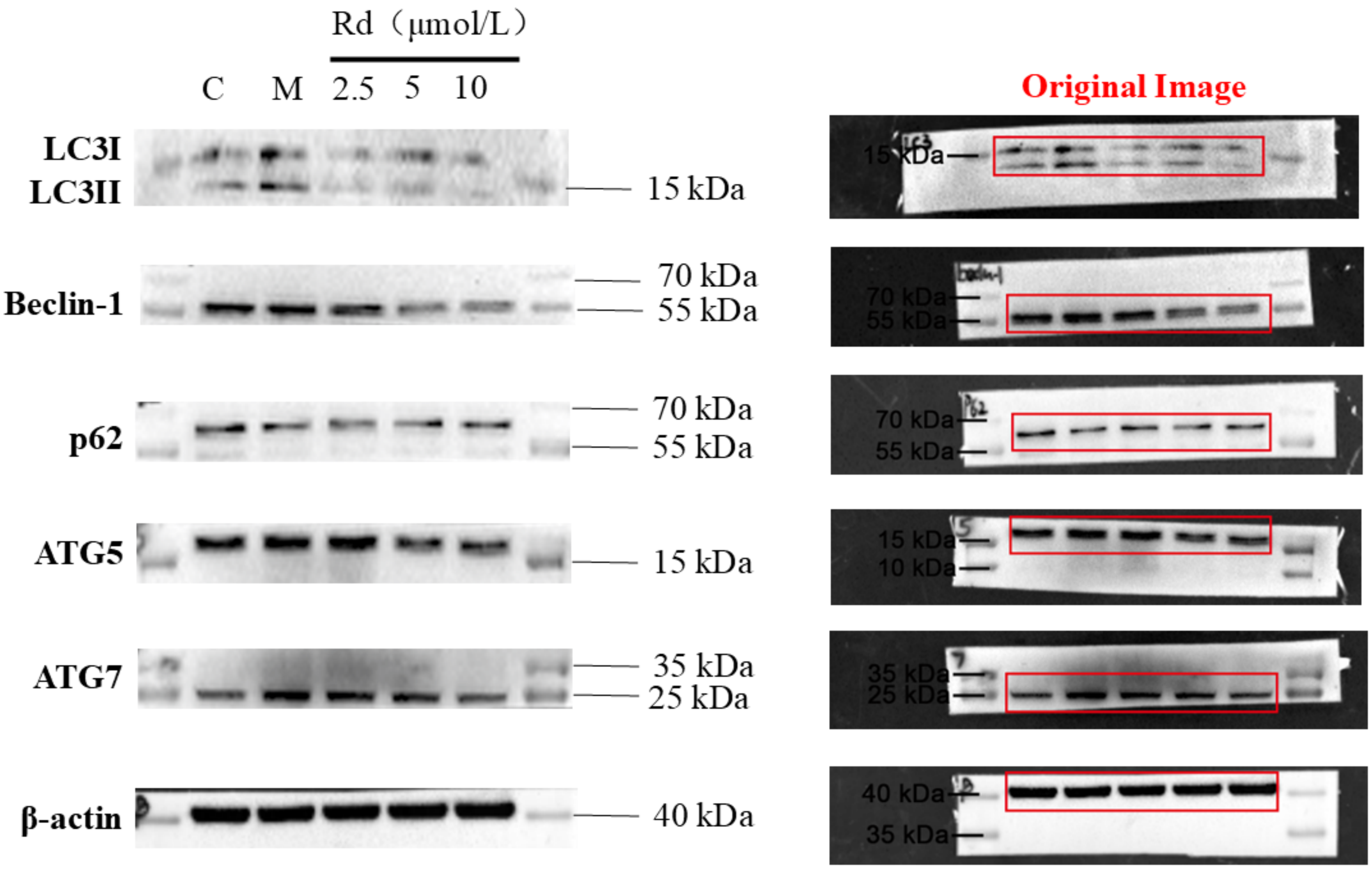

Fig7

p-AMPK: 64 kDa

AMPK: 64 kDa

p-mTOR: 289 kDa

mTOR: 289 kDa

p-ULK： 150 kDa

ULK： 150 kDa

β-actin: 43 kDa

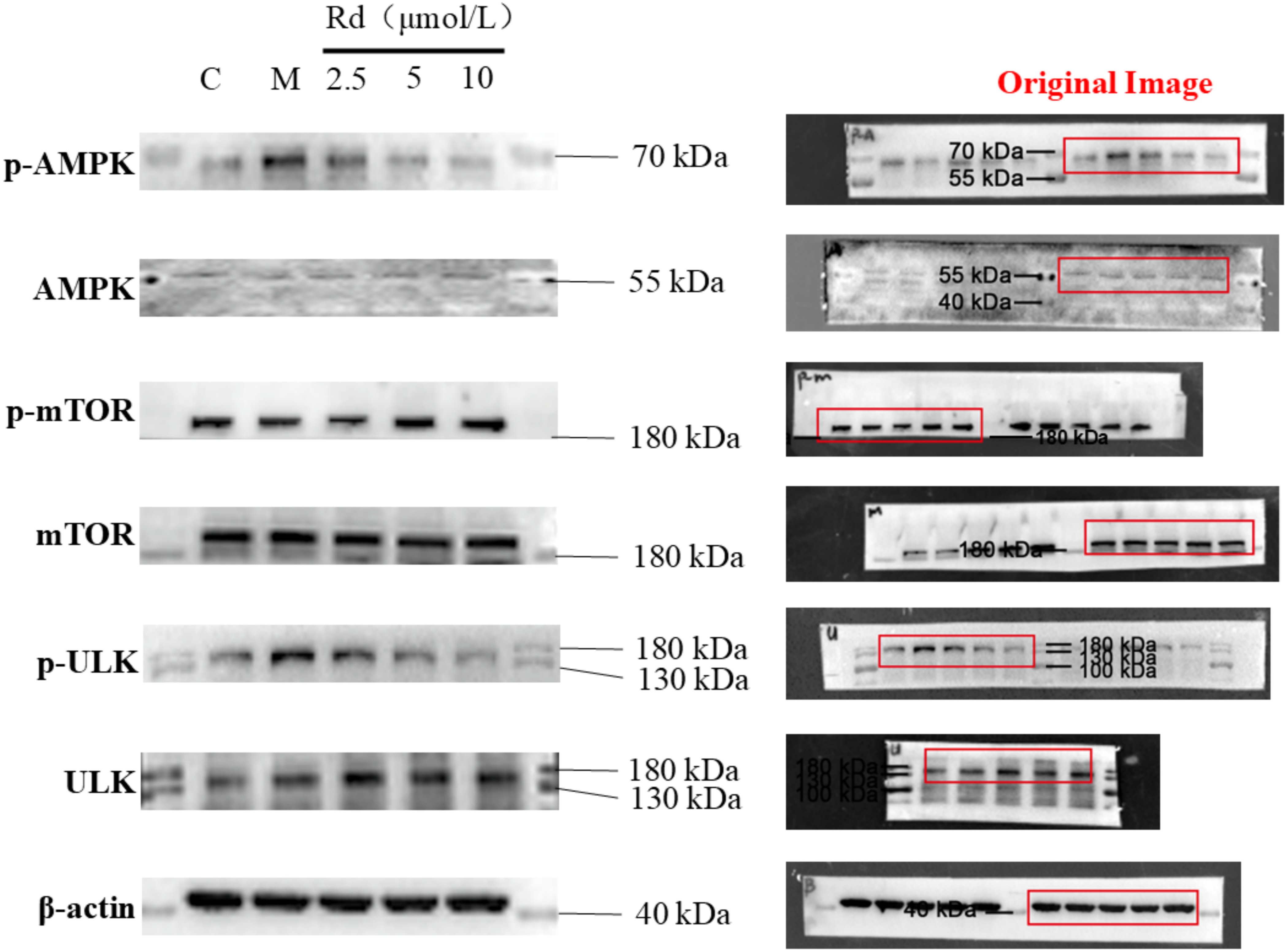

Fig8A

LC3I: 16 kDa

LC3II: 14 kDa

Beclin-1: 60 kDa

p62: 62 kDa

p-mTOR: 289 kDa

mTOR: 289 kDa

p-ULK： 150 kDa

ULK： 150 kDa

β-actin: 43 kDa

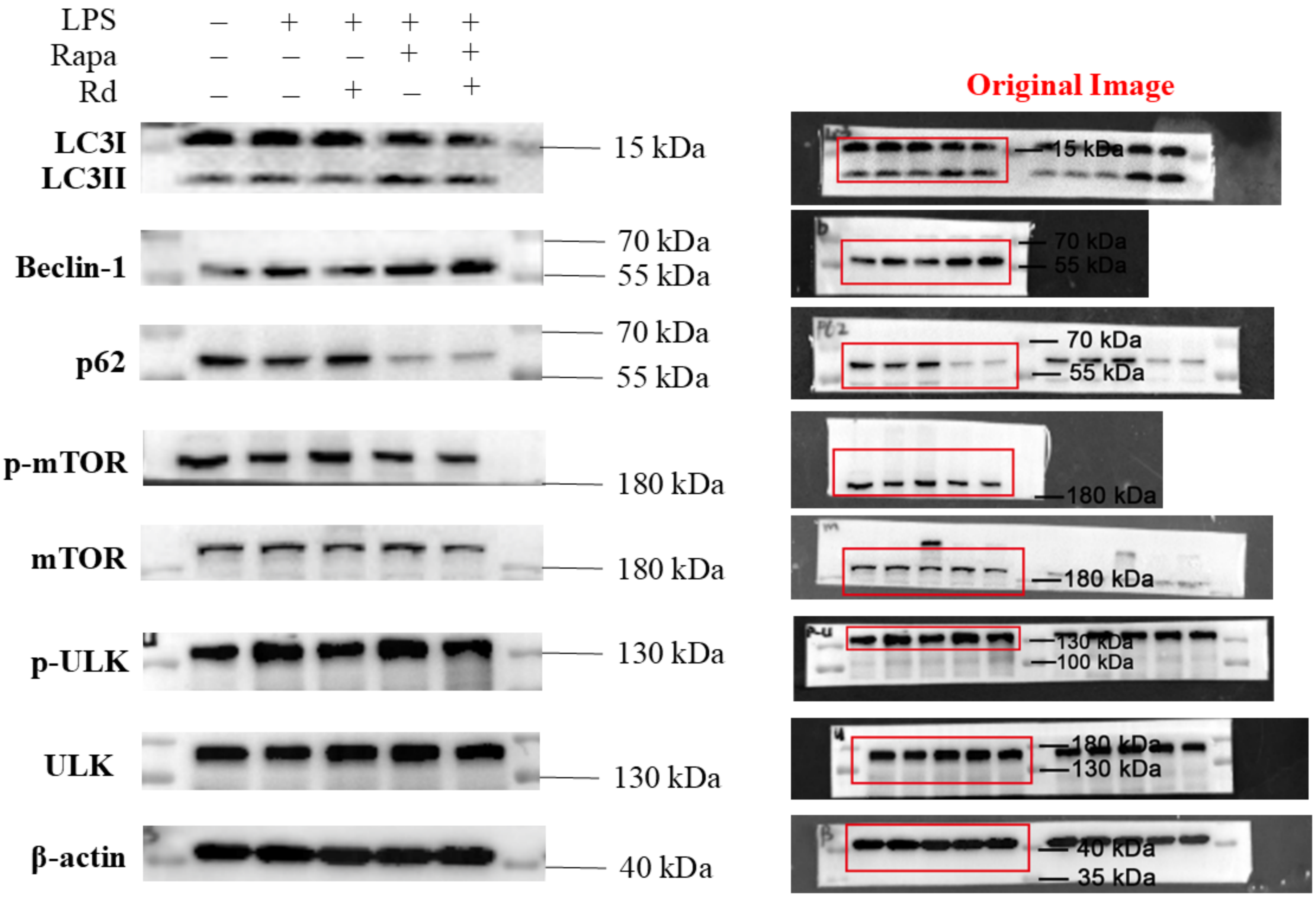

Fig8-B

NLRP3： 118 kDa

1L-1β： 22 kDa

IL-18： 18 kDa

β-actin:43 kDa

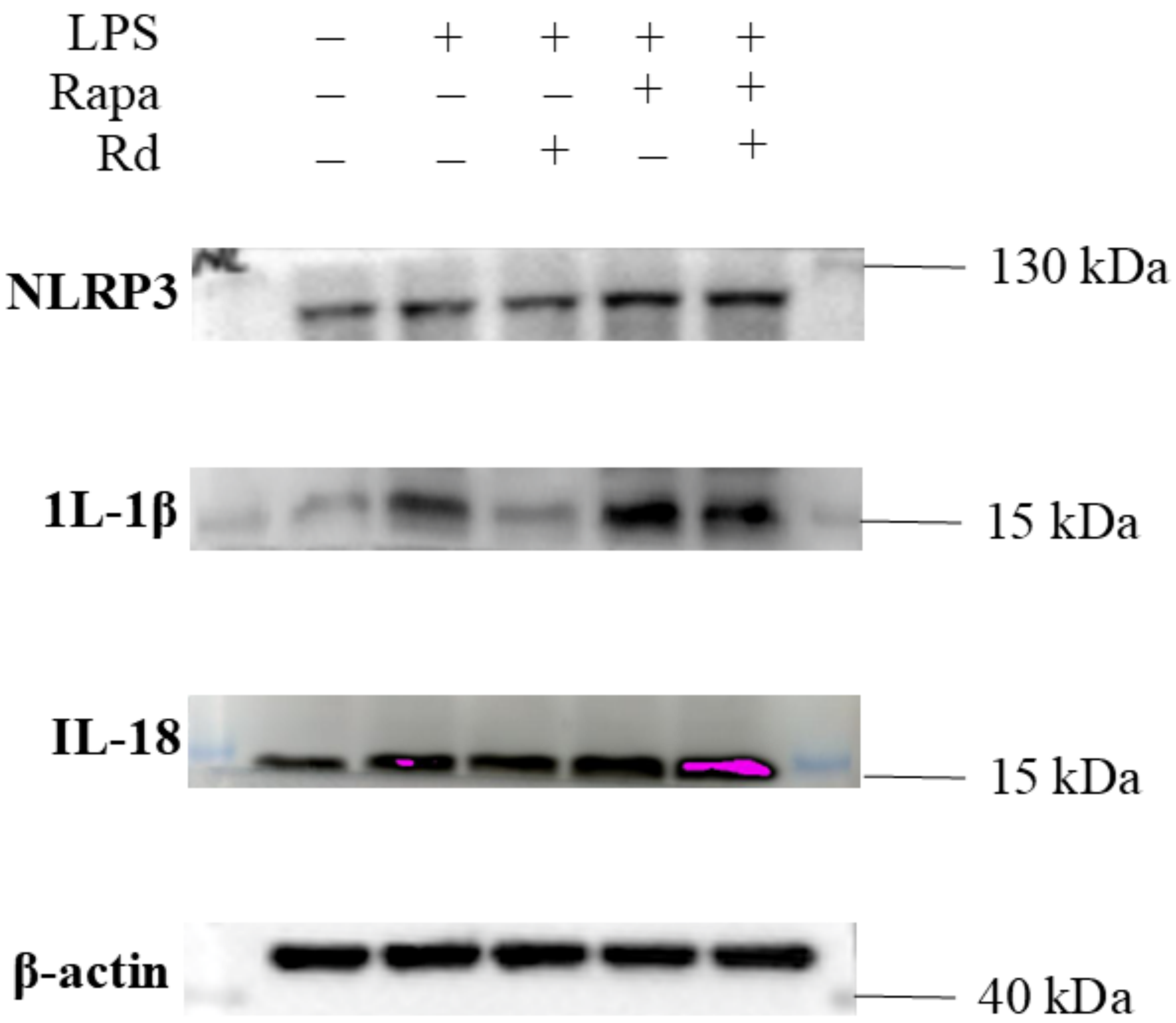

Original Image

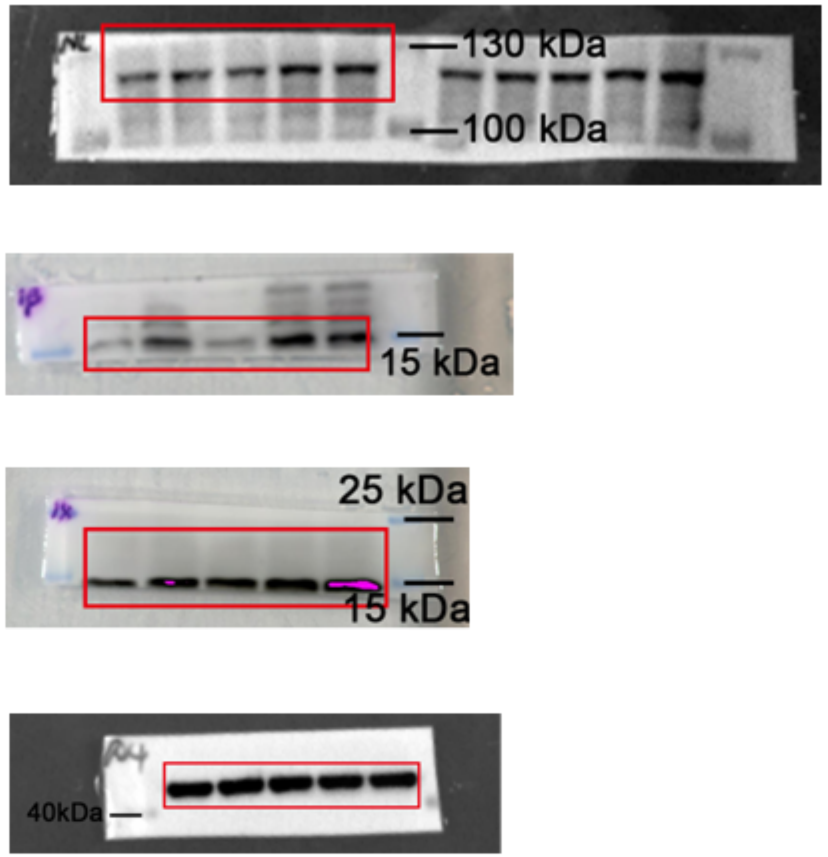

Fig9

LC3I: 16 kDa

LC3II: 14 kDa

p-AMPK: 64 kDa

AMPK: 64 kDa

p-ULK： 150 kDa

ULK： 150 kDa

β-actin: 43 kDa

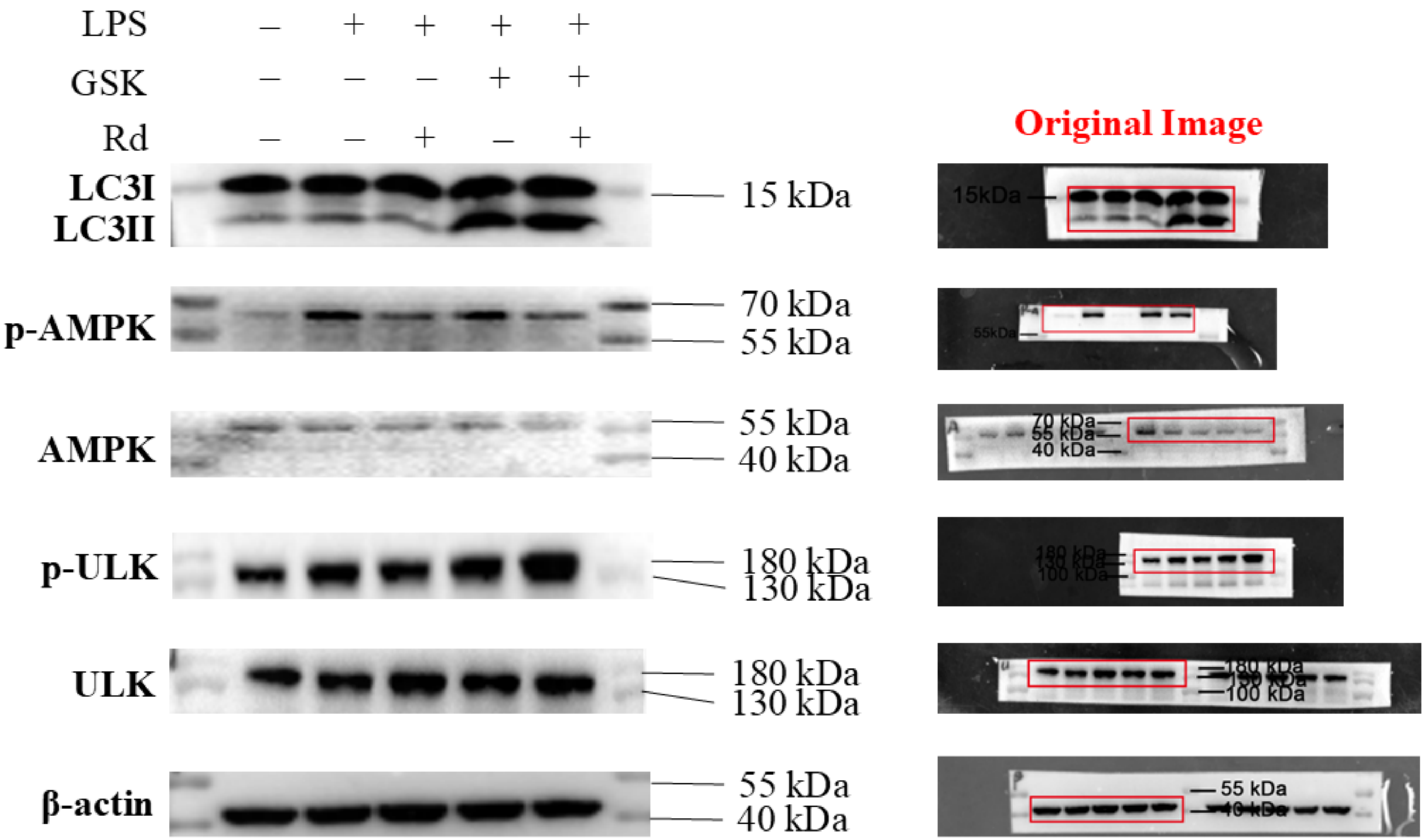

Supplement: Supplementary file 3 — Supplementary Material 3 [file 41598_2025_87991_MOESM3_ESM.pdf]
